# Supplementary material for: Formation of Pre-PCTA/DT Intermediates from 2-Chlorothiophenol on Silica Clusters: A Quantum Mechanical Study
Source: Int J Mol Sci. 2024 Mar 20;25(6):3485. doi: 10.3390/ijms25063485 (PMC10970448; doi:10.3390/ijms25063485)
Supplement: Supplementary file 1 [file ijms-25-03485-s001.zip › ijms-2893407-supplementary.pdf]

Supplementary Materials

Table S1. Imaginary frequencies (cm^{-1}), zero-point energies (ZPE, a.u.) and total energies (without ZPE, a.u.) for the transition states involved in the formation of pre-PCTA/DT intermediates from 2-CTP on $(SiO_2)_3$ and $(SiO_2)_3O_2H_4$ clusters.

**TS**	***v***	**ZPE**	**Total energy**
TS1	−1422i	0.12158	−2410.43830
TS2	−1253i	0.12179	−2410.41998
TS3	−1013i	0.12246	−2410.46239
TS4	−311i	0.17579	−2563.49883
TS5	−1078i	0.17344	−2563.50360
TS6	−1349i	0.17428	−2563.49538
TS7	−1167i	0.21816	−3500.64958
TS8	−1179i	0.21957	−3500.65038
TS9	−979i	0.21890	−3501.10248
TS10	−1328i	0.20501	−3499.98845
TS11	−408i	0.22117	−3500.57752
TS12	−394i	0.22058	−3500.54372
TS13	−1468i	0.22404	−3501.12588
TS14	−422i	0.20874	−3500.00017
TS15	−1534i	0.20470	−3499.98277
TS16	−350i	0.22053	−3500.57964
TS17	−394i	0.22048	−3500.55636
TS18	−492i	0.21976	−3500.56959
TS19	−501i	0.21925	−3500.55657
TS20	−1575i	0.21290	−3500.51817
TS21	−423i	0.22027	−3500.59741
TS22	−474i	0.24514	−3577.11994
TS23	−477i	0.24520	−3577.10508
TS24	−1528i	0.23845	−3577.06674
TS25	−432i	0.24648	−3577.15249
TS26	−501i	0.22843	−2854.45159
TS27	−392i	0.22742	−2854.53260
TS28	−1180i	0.21939	−2854.53178
TS29	−543i	0.22791	−2854.51584

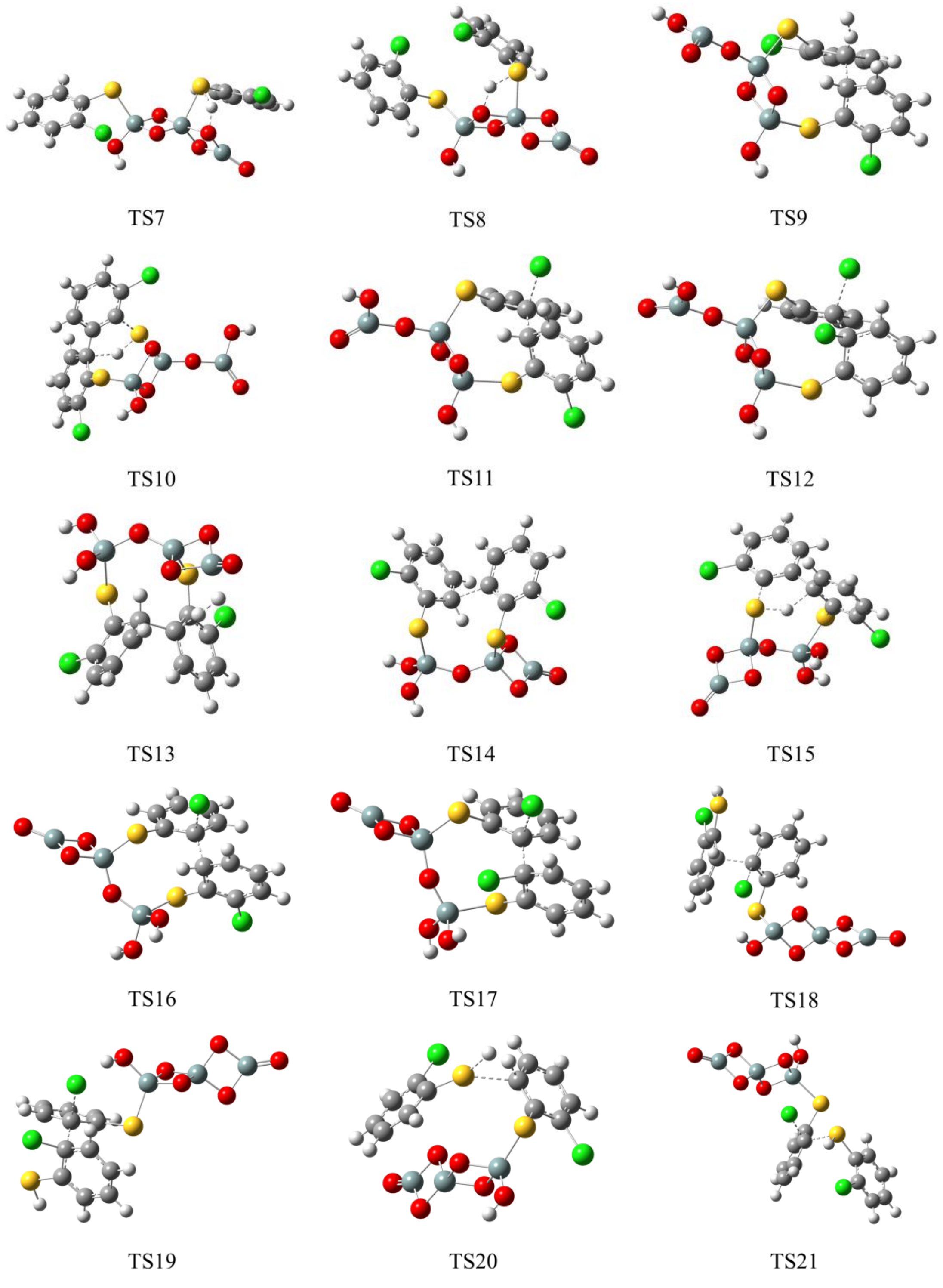

Figure S1. The structures of key transition states involved in formation of pre-PCTA/DT intermediates from the coupling of two adsorbed 2-chlorothiophenolates via the L-H mechanism and the condensation of adsorbed 2-chlorothiophenolate with gas-phase 2-CTP via the E-R mechanism on $(SiO_2)_3$ cluster.

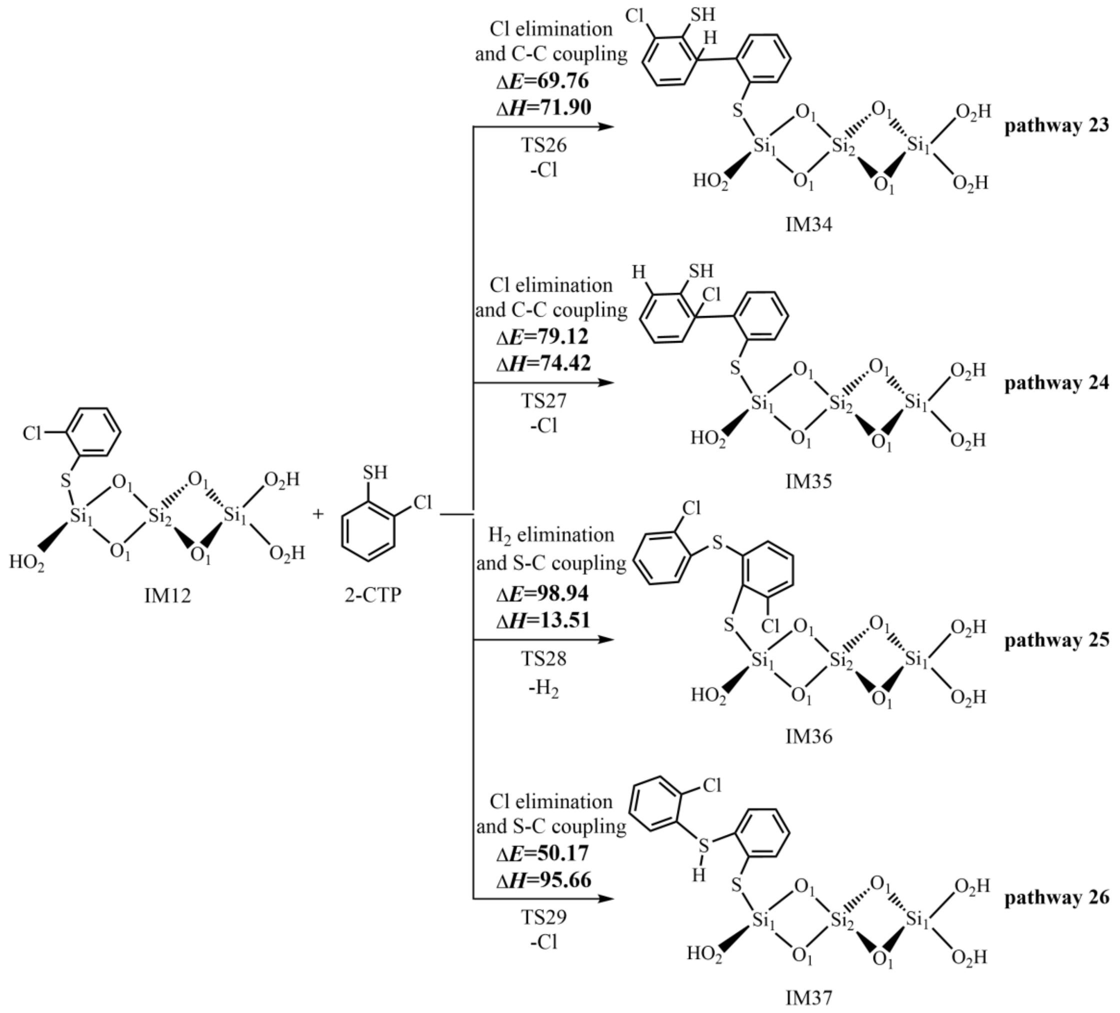

Figure S2. Formation routes of pre-PCTA/DT intermediates embedded with the potential barriers Δ*E* (kcal/mol) and reaction heats Δ*H* (kcal/mol) from the condensation of adsorbed 2-chlorothiophenolate on $(SiO_2)_3O_2H_4$ cluster with gas-phase 2-CTP via the E-R mechanism.

Table S2. CVT/SCT rate constants for crucial elementary reactions involved in the formation of pre-PCTA/DT intermediates from 2-CTP on $(SiO_2)_3$ and $(SiO_2)_3O_2H_4$ clusters over the temperature range of 600−1200 K (units are s^{-1} and cm^3 $molecule^{-1}$ s^{-1} for unimolecular and bimolecular reactions, respectively).

T(K)	CVT/SCT Rate Constants			
	TS1	TS2	TS3	TS4
600	7.64×10^{4}	1.28×10^{7}	6.54×10^{10}	7.88×10^{1}
700	1.05×10^{6}	6.11×10^{7}	1.64×10^{11}	2.15×10^{3}
800	7.62×10^{6}	1.99×10^{8}	3.28×10^{11}	2.56×10^{4}
900	3.59×10^{7}	5.02×10^{8}	5.67×10^{11}	1.76×10^{5}
1000	1.25×10^{8}	1.06×10^{9}	8.84×10^{11}	8.24×10^{5}
1100	3.48×10^{8}	1.96×10^{9}	1.28×10^{12}	2.91×10^{6}
1200	8.21×10^{8}	3.28×10^{9}	1.74×10^{12}	8.36×10^{6}
	TS5	TS6	TS7	TS8
600	2.39×10^{1}	1.40×10^{0}	1.37×10^{9}	1.67×10^{8}
700	4.74×10^{2}	3.77×10^{1}	5.02×10^{9}	6.04×10^{8}
800	4.50×10^{3}	4.49×10^{2}	1.35×10^{10}	1.60×10^{9}
900	2.61×10^{4}	3.11×10^{3}	2.93×10^{10}	3.42×10^{9}
1000	1.07×10^{5}	1.47×10^{4}	5.48×10^{10}	6.33×10^{9}
1100	3.41×10^{5}	5.25×10^{4}	9.22×10^{10}	1.05×10^{10}
1200	8.99×10^{5}	1.52×10^{5}	1.43×10^{11}	1.61×10^{10}
	TS11	TS12	TS13	TS16
600	8.20×10^{-20}	3.16×10^{-27}	2.69×10^{-32}	1.04×10^{-17}
700	2.38×10^{-15}	1.15×10^{-21}	1.04×10^{-29}	1.45×10^{-13}
800	5.36×10^{-12}	1.72×10^{-17}	9.26×10^{-28}	1.87×10^{-10}
900	2.18×10^{-09}	3.07×10^{-14}	3.13×10^{-26}	4.94×10^{-8}
1000	2.67×10^{-07}	1.23×10^{-11}	5.35×10^{-25}	4.29×10^{-6}
1100	1.37×10^{-05}	1.66×10^{-09}	5.55×10^{-24}	1.66×10^{-4}
1200	3.66×10^{-04}	9.95×10^{-08}	3.96×10^{-23}	3.49×10^{-3}
	TS17	TS18	TS19	TS20
600	1.39×10^{-23}	3.83×10^{-42}	2.80×10^{-45}	4.91×10^{-53}
700	1.11×10^{-18}	2.80×10^{-38}	5.19×10^{-41}	9.73×10^{-48}
800	5.30×10^{-15}	2.35×10^{-35}	8.73×10^{-38}	9.86×10^{-44}
900	3.88×10^{-12}	4.61×10^{-33}	2.95×10^{-35}	1.37×10^{-40}
1000	7.63×10^{-10}	3.26×10^{-31}	3.22×10^{-33}	4.67×10^{-38}
1100	5.76×10^{-8}	1.10×10^{-29}	1.54×10^{-31}	5.75×10^{-36}
1200	2.12×10^{-6}	2.10×10^{-28}	3.97×10^{-30}	3.27×10^{-34}
	TS21	TS22	TS23	TS24
600	1.85×10^{-35}	2.67×10^{-40}	6.42×10^{-44}	1.29×10^{-57}
700	1.73×10^{-32}	7.37×10^{-37}	5.79×10^{-40}	7.97×10^{-52}
800	3.11×10^{-30}	2.97×10^{-34}	5.70×10^{-37}	1.92×10^{-47}
900	1.84×10^{-28}	3.30×10^{-32}	1.27×10^{-34}	5.23×10^{-44}
1000	4.96×10^{-27}	1.48×10^{-30}	9.98×10^{-33}	3.09×10^{-41}
1100	7.58×10^{-26}	3.43×10^{-29}	3.65×10^{-31}	5.95×10^{-39}
1200	7.53×10^{-25}	4.82×10^{-28}	7.51×10^{-30}	4.93×10^{-37}

Table S2. *Cont.*

T(K)	CVT/SCT Rate Constants
	TS25
600	1.25×10^{-39}
700	3.18×10^{-36}
800	1.21×10^{-33}
900	1.28×10^{-31}
1000	5.53×10^{-30}
1100	1.24×10^{-28}
1200	1.70×10^{-27}

Table S3. The Gibbs free energy ΔG (kcal/mol) and free energy barrier Δ$G^{\neq}$ (kcal/mol) for the crucial elementary reactions involved in the formation of pre-PCTA/DT intermediates from 2-CTP on $(SiO_2)_3$ and $(SiO_2)_3O_2H_4$ clusters.

**Reactions**	Δ$G^{\neq}$ (kcal/mol)	ΔG (kcal/mol)
2-CTP+ $(SiO_2)_3$ → IM1		-10.17
2-CTP+ $(SiO_2)_3$ → IM3		10.46
2-CTP+ $(SiO_2)_3$ → IM5		-8.82
IM1 → IM2 via TS1	22.33	-4.21
IM3 → IM4 via TS2	14.75	-24.56
IM5 → IM6 via TS3	6.86	-43.55
2-CTP+ $(SiO_2)_3$ → IM7		11.60
2-CTP+ $(SiO_2)_3$ → IM9		7.33
2-CTP+ $(SiO_2)_3$ → IM11		10.38
IM7 → IM8 via TS4	28.81	-12.54
IM9 → IM10 via TS5	28.48	-10.11
IM11 → IM12 via TS6	31.67	7.99
2-CTP+IM6→ IM13		21.76
IM13 → IM14 via TS7	10.81	-28.35
IM13 → IM15 via TS8	12.05	-23.37
IM14 → IM16+ H_2 via TS9	75.34	-8.72
IM16 → IM17 via TS10	39.57	6.31
IM14 → IM18 + Cl via TS11	86.59	68.04
IM14 → IM19+ Cl via TS12	107.26	83.58
IM15 → IM20 + H_2 via TS13	59.52	8.74
IM20 → IM21 via TS14	13.56	-19.99
IM21 → IM22 via TS15	42.38	14.73
IM15 → IM23 + Cl via TS16	80.64	64.76
IM15 → IM24 + Cl via TS17	95.94	74.14
IM6 + 2-CTP → IM25 + Cl via TS18	84.24	74.08
IM6 + 2-CTP → IM26 + Cl via TS19	92.43	77.15
IM6 + 2-CTP → IM27 + H_2 via TS20	113.41	18.65
IM6 + 2-CTP → IM28 + Cl via TS21	66.49	103.07
IM29 + 2-CP → IM30 + Cl via TS22	77.63	73.46
IM29 + 2-CP → IM31 + Cl via TS23	87.40	77.69
IM29 + 2-CP → IM32 + H_2 via TS24	123.97	30.97
IM29 + 2-CP → IM33 + Cl via TS25	76.37	113.49
IM12 + 2-CTP → IM34 + Cl via TS26	81.78	75.57
IM12 + 2-CTP → IM35 + Cl via TS27	93.23	78.15
IM12 + 2-CTP → IM36 + H_2 via TS28	112.49	20.34
IM12 + 2-CTP → IM37 + Cl via TS29	62.27	96.47

Table S4. Cartesian coordinates for transition states involved in the formation of pre-PCTA/DT intermediates from 2-CTP on $(SiO_2)_3$ and $(SiO_2)_3O_2H_4$ clusters.

TS1

Si	2.350985	−0.35233	0.00604
O	3.76076	−0.70875	−0.789845
O	3.000228	1.104886	0.469246
Si	0.24903	−1.58462	0.330237
O	0.919502	−0.31337	−0.840325
O	1.724152	−1.36615	1.112448
O	−0.402203	−2.88875	−0.065733
Si	4.406159	0.747801	−0.333675
O	5.708896	1.456368	−0.558342
C	−3.074454	−1.31689	−0.051338
C	−2.541328	−0.06545	0.216532
C	−3.279456	1.078355	−0.070638
C	−4.540001	0.970277	−0.627793
C	−5.066514	−0.27917	−0.89628
C	−4.335578	−1.41745	−0.609871
H	−2.491246	−2.20276	0.149622
H	−5.095653	1.867537	−0.846286
H	−6.05024	−0.35772	−1.331444
H	−4.739726	−2.3941	−0.822393
Cl	−2.648282	2.647552	0.258344
H	0.032155	0.383967	−0.226437
S	−0.961726	0.14068	1.016587

TS2

C	1.390815	−2.52509	0.35851
C	1.377702	−1.16624	0.639694
C	2.347131	−0.3554	0.067214
C	3.317907	−0.88292	−0.761971
C	3.322978	−2.23873	−1.026925
C	2.358564	−3.05884	−0.469432
H	0.628263	−3.15764	0.784372
H	4.055392	−0.22816	−1.196556
H	4.079533	−2.64911	−1.676234
H	2.352267	−4.11578	−0.681085
Cl	2.388241	1.352838	0.362229
H	0.120519	0.922287	1.669738
Si	−1.220522	0.292873	0.060018
O	−2.85665	0.496175	0.353125
O	−1.641586	−1.22724	−0.632429

Si	0.035328	2.391428	−0.419151
O	−0.407673	1.014011	−1.207015
O	−0.650267	1.816754	0.970417
O	0.560316	3.732633	−0.852449
Si	−3.223138	−0.95996	−0.340046
O	−4.503992	−1.72157	−0.541748
S	0.0743	−0.58958	1.698038

TS3

C	1.330356	1.653275	0.533063
C	2.09716	0.498461	0.482613
C	3.37351	0.541059	−0.064253
C	3.863689	1.72942	−0.577569
C	3.090738	2.873423	−0.530633
C	1.824668	2.838355	0.027145
H	0.340819	1.618891	0.961757
H	4.851933	1.74488	−1.007213
H	3.483239	3.794481	−0.931357
H	1.219182	3.729329	0.067147
Cl	4.366254	−0.86168	−0.116232
H	1.754863	−1.97871	0.192785
Si	−2.229439	−0.44212	−0.153029
O	−3.68871	−0.45447	−0.94958
O	−2.807104	0.779763	0.825909
Si	−0.159905	−1.56236	−0.266736
O	−1.626877	−1.8091	0.522285
O	−0.823506	−0.18154	−0.960919
O	0.792122	−2.60534	−0.88431
Si	−4.253255	0.758503	0.022824
O	−5.535945	1.530136	0.131045
S	1.447467	−0.98101	1.222022

TS4

Si	−1.697393	−0.58373	−0.183907
O	−0.928265	−1.33294	−1.339048
O	−0.591288	−0.82615	1.048978
Si	−3.698483	0.647998	0.13944
O	−2.143982	1.026116	−0.312948
O	−3.257503	−0.94846	0.279042
Si	0.542321	−1.83229	0.430977
O	−4.786314	1.005037	−1.001049
H	−5.638935	1.322696	−0.717025

O	−4.245471	1.311931	1.518895
H	−3.817551	1.078022	2.338132
O	1.72437	−1.90406	1.551133
H	1.536727	−1.5066	2.397417
O	0.221944	−3.35083	−0.023009
H	−0.423972	−3.36552	−0.733952
C	0.960357	1.62096	−0.817109
C	1.988758	0.710069	−0.63447
C	3.146859	1.090055	0.031899
C	3.270037	2.380428	0.514696
C	2.239648	3.284135	0.335065
C	1.085974	2.9072	−0.329858
H	0.055673	1.327305	−1.326906
H	4.174368	2.663558	1.028199
H	2.342709	4.287008	0.718439
H	0.274628	3.603406	−0.466234
Cl	4.439418	−0.02027	0.261511
H	0.624389	−0.8176	−1.878412
S	1.828368	−0.95612	−1.224612

TS5

Si	1.182015	0.241215	−0.092518
O	2.654206	0.368294	−0.862035
O	1.839357	−1.09793	0.752308
Si	−0.084296	2.358106	0.275704
O	0.317243	1.545236	−1.108679
O	0.651157	1.181742	1.17396
Si	3.290457	−0.90969	0.001517
O	−1.681445	2.469345	0.509743
H	−2.04449	3.32627	0.716154
O	0.522066	3.852306	0.44473
H	1.459975	3.99116	0.342314
O	4.533496	−0.49946	0.952116
H	5.337679	−1.00388	0.86561
O	3.814646	−2.16378	−0.893163
H	3.214603	−2.59106	−1.497545
C	−1.285749	−1.55877	1.262504
C	−1.634876	−1.1192	−0.006859
C	−2.966075	−0.84619	−0.296446
C	−3.936966	−1.00568	0.675259
C	−3.581648	−1.44148	1.937311
C	−2.258187	−1.71782	2.230847
H	−0.24931	−1.76294	1.486079

H	−4.963413	−0.78421	0.432256
H	−4.343957	−1.56241	2.690806
H	−1.975109	−2.0524	3.215929
Cl	−3.441936	−0.3058	−1.861437
H	−0.457812	0.491012	−1.556306
S	−0.382781	−0.97618	−1.262765

TS6

Si	1.525452	−0.5164	−0.167579
O	0.111895	−0.54463	−1.002965
O	1.094272	−1.87161	0.637992
Si	3.42862	0.887583	−0.171057
O	2.960411	−0.4807	−1.001061
O	1.989188	0.836631	0.669871
Si	−0.394029	−1.97356	−0.221836
O	3.606637	2.217505	−1.067424
H	4.452995	2.656017	−1.054946
O	4.782835	0.730633	0.707736
H	4.907564	−0.04322	1.250229
O	−0.425042	−3.23047	−1.252672
H	−0.272754	−3.02582	−2.171681
O	−1.211296	−2.64369	1.180924
H	−0.685974	−2.65958	1.984065
C	−3.361648	1.206589	−1.248836
C	−2.676608	0.504051	−0.263519
C	−2.043191	1.23379	0.734433
C	−2.082014	2.615596	0.749341
C	−2.765014	3.293662	−0.241259
C	−3.408467	2.586323	−1.241537
H	−3.858765	0.649883	−2.027633
H	−1.570565	3.146823	1.535337
H	−2.791526	4.371689	−0.227808
H	−3.94507	3.10656	−2.019036
Cl	−1.181823	0.438133	2.003178
H	−2.212283	−1.92102	0.960946
S	−2.688924	−1.25286	−0.396739

TS7

C	2.964675	2.127015	−0.415837
C	3.134081	1.138477	0.546067
C	4.39064	0.569846	0.73411
C	5.458103	0.97452	−0.051562

C	5.277983	1.954737	−1.006305
C	4.033616	2.536806	−1.185092
H	1.98007	2.541699	−0.572773
H	6.419419	0.510084	0.094399
H	6.113951	2.259431	−1.615528
H	3.889135	3.297592	−1.935053
Cl	4.667619	−0.62748	1.941432
H	2.062726	−0.86221	1.382929
Si	0.765539	−0.35841	−0.345227
O	1.857144	−1.76192	0.420592
O	1.792695	−0.64414	−1.640229
Si	−1.556601	0.179151	−0.641554
O	−0.125481	0.980086	−0.884048
O	−0.669756	−1.14597	−0.102584
O	−2.501286	−0.15953	−1.910323
Si	2.735464	−1.79642	−0.956674
O	3.941201	−2.55396	−1.444317
S	1.708824	0.617807	1.482298
H	−2.284781	−0.90519	−2.465079
C	−4.791248	−0.55824	0.38598
C	−6.117386	−0.87649	0.155025
C	−7.036399	0.127471	−0.081381
C	−6.626348	1.448826	−0.092356
C	−5.297839	1.759791	0.12566
C	−4.360256	0.763798	0.3695
H	−6.418396	−1.91146	0.167404
H	−8.069943	−0.1262	−0.25684
H	−7.335939	2.23941	−0.278468
H	−4.967963	2.786437	0.105443
S	−2.70453	1.273052	0.736944
Cl	−3.680117	−1.83957	0.685538

TS8

C	1.566881	2.644804	−0.554796
C	0.867534	2.061166	0.492334
C	−0.470469	2.376855	0.692137
C	−1.107765	3.262303	−0.1572
C	−0.406697	3.839159	−1.198659
C	0.927739	3.532067	−1.398491
H	2.60404	2.389441	−0.710301
H	−2.15298	3.476455	−0.005275
H	−0.910897	4.526488	−1.85934
H	1.475161	3.976031	−2.214265

Cl	−1.36446	1.661069	1.978173
H	0.778808	−0.24183	1.496196
Si	2.377184	−0.55612	0.052166
O	3.456101	−1.70834	0.63803
O	3.783714	0.315441	−0.467525
Si	0.253707	−1.53035	−0.817752
O	1.587823	−0.73432	−1.398365
O	0.838485	−1.36376	0.732046
O	0.117437	−3.08583	−1.259387
Si	4.762694	−0.85426	0.098564
O	6.246741	−1.09739	0.158323
S	1.713125	0.949257	1.597854
H	0.80697	−3.69251	−0.993237
C	−3.896034	−0.40855	−0.170945
C	−4.949881	−0.90755	0.572503
C	−4.879888	−2.18177	1.100784
C	−3.759016	−2.95701	0.86632
C	−2.711609	−2.45977	0.11517
C	−2.752348	−1.1699	−0.400981
H	−5.817758	−0.28773	0.729117
H	−5.70247	−2.56593	1.682632
H	−3.697662	−3.95973	1.258686
H	−1.863356	−3.09157	−0.091269
S	−1.455141	−0.45265	−1.375307
Cl	−4.045591	1.179997	−0.830492

TS9

C	1.390913	1.314894	1.372044
C	0.308532	1.690612	0.504456
C	0.54819	2.465986	−0.619378
C	1.817014	2.947655	−0.892804
C	2.848887	2.7157	0.019514
C	2.648228	1.953745	1.133635
H	1.039136	1.334556	2.458391
H	1.984493	3.522131	−1.788164
H	3.818868	3.151502	−0.165639
H	3.457476	1.762863	1.822291
Cl	−0.72378	2.884751	−1.699416
S	−1.325799	1.462269	1.166849
C	3.692934	−1.13857	0.200185
C	4.431741	−1.11239	1.376582
C	3.774791	−0.98154	2.599457
C	2.430065	−0.76564	2.623305

C	1.682093	−0.53848	1.419591
C	2.321643	−0.96374	0.199069
H	5.498975	−1.25134	1.3278
H	4.334591	−1.06819	3.517563
H	1.910185	−0.65867	3.563338
H	0.638298	−0.81005	1.502826
S	1.545407	−0.9195	−1.37426
Cl	4.563145	−1.42461	−1.262473
H	0.375431	1.236837	3.584082
Si	−1.950709	−0.19213	0.099634
O	−3.523614	−0.38298	0.400912
Si	−0.473199	−1.51572	−1.169239
O	−1.026735	−1.55661	0.399911
O	−1.528688	−0.27267	−1.501297
O	−0.771701	−2.79608	−2.105905
Si	−5.08167	−0.65186	0.624998
O	−5.72504	−1.6198	1.586283
H	−0.059323	−3.38194	−2.34851
O	−5.834603	0.357829	−0.382778
H	−6.790103	0.324592	−0.369726

TS10

C	1.562293	1.857717	0.180302
C	0.219727	2.189355	0.291928
C	−0.290898	3.315905	−0.324506
C	0.542915	4.142804	−1.053045
C	1.887655	3.832594	−1.151118
C	2.397363	2.705969	−0.531163
H	0.136397	5.018936	−1.532354
H	2.54001	4.473701	−1.722978
H	3.447275	2.467085	−0.608868
Cl	−1.964498	3.710431	−0.185448
S	−0.843081	1.015622	1.254499
C	3.174104	−1.52352	0.745363
C	3.633847	−1.3886	2.046792
C	3.397859	−0.20445	2.728786
C	2.672403	0.804356	2.142868
C	2.111444	0.643708	0.858524
C	2.461405	−0.5141	0.11548
H	4.188208	−2.1939	2.498385
H	3.787866	−0.07815	3.726748
H	2.482509	1.723407	2.675652
H	0.494582	0.354432	1.340554

S	2.054677	−0.62863	−1.607884
Cl	3.535591	−2.99944	−0.071289
Si	−1.444036	−0.65396	0.072742
O	−2.977336	−1.03967	0.377754
Si	0.12665	−1.45731	−1.495196
O	−0.285051	−1.84609	0.082332
O	−1.137057	−0.36047	−1.528033
O	−0.087355	−2.58071	−2.634978
Si	−4.492942	−1.51038	0.574199
O	−5.023684	−2.91953	0.655299
H	0.642012	−3.14401	−2.881907
O	−5.340434	−0.14338	0.705292
H	−6.281764	−0.24872	0.836424

TS11

C	−1.453455	1.492873	0.383493
C	−0.199732	1.52282	1.013041
C	−0.118529	1.533262	2.402878
C	−1.252915	1.510757	3.179986
C	−2.498747	1.50657	2.554831
C	−2.60321	1.500163	1.188356
H	−1.175935	1.513133	4.254819
H	−3.401101	1.506153	3.147371
H	−3.571605	1.500789	0.710323
S	1.265532	1.862083	0.058343
C	−3.369158	−1.17547	−0.949279
C	−4.051968	−0.44053	−1.919827
C	−3.474129	0.664278	−2.523215
C	−2.237357	1.076845	−2.11318
C	−1.554087	0.472629	−0.993718
C	−2.119197	−0.79626	−0.515891
H	−5.030929	−0.78011	−2.220117
H	−3.987871	1.173971	−3.321576
H	−1.746127	1.899728	−2.599655
H	−0.48616	0.413665	−1.20399
S	−1.459506	−1.67851	0.829045
Cl	−4.17256	−2.56624	−0.3272
Cl	−1.505125	3.475977	−0.735481
H	0.857666	1.577766	2.859465
Si	2.028675	−0.06687	0.067047
O	3.58766	−0.05539	−0.34299
Si	0.632708	−1.91121	0.50886
O	1.128649	−1.13845	−0.87084

O	1.641702	−0.96361	1.417169
O	0.969286	−3.4748	0.71047
Si	5.167168	−0.13883	−0.570719
O	6.232081	−0.5169	0.427194
H	0.31942	−4.14466	0.514629
O	5.384489	0.282832	−2.112855
H	6.291156	0.296593	−2.416567

TS12

C	1.860856	−0.99916	0.537975
C	0.562311	−1.00583	1.096391
C	0.407933	−0.82215	2.475205
C	1.474115	−0.65525	3.320628
C	2.759591	−0.70348	2.780616
C	2.943531	−0.86066	1.437111
H	1.317706	−0.52006	4.378104
H	3.623661	−0.60204	3.419758
H	3.944029	−0.87299	1.03349
S	−0.870662	−1.67766	0.255831
C	3.702779	1.783236	−0.51797
C	4.791224	1.058477	−0.979519
C	4.637187	−0.22494	−1.508043
C	3.403677	−0.78857	−1.521483
C	2.229464	−0.17077	−0.897847
C	2.435283	1.244684	−0.496921
H	5.76681	1.520257	−0.961431
H	5.477523	−0.74535	−1.937299
H	3.2285	−1.75119	−1.968912
S	1.322259	2.278734	0.32512
Cl	1.99584	−3.06776	−0.358447
H	−0.594495	−0.85668	2.871638
H	3.849675	2.788477	−0.156497
Cl	0.926165	−0.28833	−2.0857
Si	−1.958727	0.075276	0.085112
O	−3.531489	−0.24115	−0.081217
Si	−0.815275	2.120806	0.1022
O	−1.424429	1.162588	−1.087241
O	−1.552163	1.198425	1.256872
O	−1.261334	3.666865	0.188884
Si	−5.094268	−0.51389	−0.255171
O	−6.20963	−0.16857	0.699345
H	−0.768853	4.362436	−0.237667
O	−5.235144	−1.25211	−1.683322

H	−6.12263	−1.49921	−1.939303

TS13

C	−0.226741	1.298752	0.763182
C	−0.70566	1.509949	−0.579124
C	−0.800736	2.821217	−1.063576
C	−0.504207	3.902168	−0.267459
C	−0.089252	3.726857	1.050913
C	0.089884	2.463211	1.526829
H	−1.537869	1.025016	1.167143
H	−0.62187	4.892792	−0.67765
H	0.117978	4.584667	1.669184
H	0.473354	2.314684	2.52223
Cl	−1.342658	3.113585	−2.666159
Si	−1.834857	−1.42719	−0.260392
O	−3.46164	−1.28041	−0.172898
O	−1.713469	−1.32245	1.37734
Si	0.77163	−2.62443	−0.952538
O	−0.869805	−2.55404	−0.895511
O	1.220006	−3.18573	0.50372
Si	−3.29003	−0.67072	1.430809
O	−4.316209	−0.6798	2.543521
S	−1.382054	0.277086	−1.553628
H	2.105173	−3.035	0.825279
C	3.071224	0.469491	0.778542
C	3.195329	0.765307	2.140291
C	2.159886	0.39953	3.03125
C	0.957297	0.002726	2.562874
C	0.663866	0.036472	1.105591
C	1.902102	−0.03083	0.264455
H	4.131634	1.149867	2.509145
H	2.351173	0.429576	4.093228
H	0.155105	−0.30606	3.216295
H	0.094445	−0.85487	0.907594
S	1.83451	−0.78619	−1.321934
Cl	4.482666	0.616735	−0.203887
O	1.071763	−3.55154	−2.243484
H	1.963791	−3.78224	−2.486248
H	−2.62989	0.827334	1.219126

TS14

C	−0.171725	1.76653	0.308148

C	−1.202252	1.522858	−0.578267
C	−2.122377	2.55445	−0.791168
C	−1.983493	3.777095	−0.158995
C	−0.924014	3.987945	0.698916
C	−0.014312	2.971071	0.946244
H	−2.708269	4.551235	−0.349416
H	−0.810482	4.944811	1.185155
H	0.803105	3.12818	1.630858
Cl	−3.461532	2.352675	−1.858879
Si	−1.517461	−1.43506	−0.173354
O	−2.975144	−2.26972	−0.12406
O	−1.844686	−0.87116	1.378133
Si	1.341077	−2.52141	−0.515408
O	−0.283782	−2.46455	−0.265541
O	1.919709	−2.8367	0.969689
Si	−3.274748	−1.69574	1.390074
O	−4.38921	−1.85043	2.386174
S	−1.314367	0.069425	−1.598507
H	2.863865	−2.84662	1.105943
C	3.446109	1.003973	0.339382
C	3.629958	1.675165	1.539393
C	2.68563	1.557169	2.554531
C	1.528679	0.853086	2.337727
C	1.265951	0.29823	1.069678
C	2.312085	0.23628	0.112837
H	4.533472	2.243852	1.686751
H	2.875226	2.016615	3.512129
H	0.782081	0.754014	3.109501
H	0.490525	−0.44622	1.032814
S	2.196186	−0.76662	−1.332432
Cl	4.700508	1.08914	−0.838839
O	1.644536	−3.63592	−1.653822
H	1.041816	−4.36606	−1.771008

TS15

C	1.093679	2.04522	0.154418
C	−0.278756	2.185927	0.264871
C	−0.947507	3.228697	−0.347063
C	−0.240003	4.158657	−1.085788
C	1.134277	4.037402	−1.189314
C	1.799334	2.998162	−0.56407
H	−0.767468	4.965006	−1.569603
H	1.688348	4.759167	−1.768647

H	2.872488	2.908614	−0.641736
Cl	−2.658904	3.380677	−0.203804
Si	−1.964537	−0.70523	0.097493
O	−3.617607	−0.53056	−0.082865
O	−2.271423	−2.20554	0.847079
Si	0.334913	−1.65902	−1.35291
O	−1.13425	−0.89965	−1.266755
O	0.5486	−2.37859	0.094769
Si	−3.893347	−1.98949	0.635408
O	−5.122271	−2.78587	0.97156
S	−1.152372	0.882112	1.247154
H	−0.119156	−2.9717	0.4416
C	3.361856	−0.92601	0.683893
C	3.768862	−0.71976	1.99387
C	3.240841	0.343224	2.710249
C	2.305574	1.17282	2.139869
C	1.819032	0.929649	0.838273
C	2.421904	−0.10692	0.07953
H	4.502792	−1.37693	2.429107
H	3.57756	0.524147	3.719148
H	1.907088	2.010297	2.691873
H	0.265644	0.328475	1.204528
S	1.94754	−0.33165	−1.612933
Cl	4.074202	−2.24194	−0.171131
O	0.192844	−2.62217	−2.64994
H	0.969857	−3.06412	−2.983005

TS16

C	1.175361	−1.63803	0.077063
C	−0.192824	−1.51823	0.380077
C	−0.650298	−1.83684	1.653492
C	0.226621	−2.23581	2.638466
C	1.578663	−2.36336	2.335097
C	2.047919	−2.08153	1.076095
H	−0.140285	−2.46939	3.624481
H	2.275451	−2.6834	3.094675
H	3.098705	−2.17819	0.848815
Si	−2.5513	0.217321	−0.236263
O	−3.409518	−0.09837	1.177598
O	−4.016891	0.245358	−1.059786
Si	−0.407003	2.298008	−0.560782
O	−1.879077	1.692389	−0.186943
O	0.122895	1.922858	−2.043776

Si	−4.829395	−0.07644	0.336071
O	−6.274182	−0.25763	0.706839
S	−1.317843	−1.28834	−0.978335
H	0.040998	2.571226	−2.740055
C	3.492434	0.915174	0.272117
C	4.515445	0.211425	−0.364726
C	4.239102	−0.69007	−1.382349
C	2.940195	−0.97044	−1.700152
C	1.841111	−0.42999	−0.939273
C	2.175757	0.690315	−0.053686
H	5.534736	0.421642	−0.080602
H	5.048696	−1.15922	−1.917393
H	2.701981	−1.66824	−2.482463
H	0.956319	−0.25723	−1.555576
S	0.940003	1.475339	0.872631
Cl	3.95438	2.07413	1.455261
O	−0.498433	3.911227	−0.427485
H	−1.148962	4.30937	0.145242
Cl	1.087949	−3.24315	−1.568995
H	−1.709275	−1.79203	1.85634

TS17

C	1.570631	−1.2279	0.271482
C	0.190724	−1.46326	0.447168
C	−0.273272	−1.87736	1.701292
C	0.569172	−2.08479	2.764465
C	1.938198	−1.93124	2.564099
C	2.420994	−1.51574	1.352689
H	0.177678	−2.40549	3.715725
H	2.633524	−2.1317	3.364908
H	3.483864	−1.39134	1.216872
Si	−2.298389	−0.24244	−0.404416
O	−3.235804	−0.50467	0.977225
O	−3.708881	−0.33369	−1.312244
Si	−0.706863	2.45604	0.029015
O	−1.667574	1.238427	−0.393648
O	−0.182087	3.378857	−1.190724
Si	−4.594548	−0.59835	0.052804
O	−6.05232	−0.83288	0.335258
S	−0.94244	−1.71867	−0.91675
H	0.367319	2.996469	−1.871214
C	3.484675	1.540433	0.931266
C	4.6598	1.002795	0.424471

C	4.650078	0.131896	−0.668314
C	3.463009	−0.24172	−1.211409
C	2.180174	0.110944	−0.601564
C	2.258637	1.194653	0.413093
H	5.600892	1.311114	0.854382
H	5.575019	−0.20248	−1.10868
H	3.416035	−0.85059	−2.096584
S	0.900868	1.766977	1.298654
O	−1.543764	3.382624	1.058051
H	−1.292673	4.293917	1.183873
Cl	2.063498	−2.76423	−1.404856
H	−1.327058	−2.08761	1.802794
H	3.525399	2.255612	1.736735
Cl	1.074671	0.550341	−1.919655

TS18

C	0.12583	−2.6867	0.115698
C	0.811283	−2.56276	1.312525
C	1.364486	−1.3559	1.686355
C	1.244884	−0.25719	0.845186
C	0.454878	−0.32868	−0.304823
C	−0.062187	−1.56633	−0.669014
C	2.823725	0.714237	0.784992
C	3.874998	−0.16678	0.30685
C	2.571141	1.895554	0.020607
C	4.321901	−0.01967	−0.988492
C	3.100133	2.053185	−1.233615
C	3.92504	1.070708	−1.755141
H	4.312814	1.16002	−2.757765
H	1.91716	2.634494	0.448332
H	−0.302169	−3.63065	−0.179737
H	2.856449	2.919112	−1.82636
H	−0.65977	−1.62236	−1.566115
H	0.917086	−3.40882	1.974053
H	2.822253	0.871467	1.857051
Cl	5.458443	−1.11362	−1.686893
S	4.452711	−1.33812	1.423425
H	5.207925	−2.02272	0.561288
H	1.867241	−1.25631	2.633979
S	−0.037804	1.1041	−1.208471
H	−1.077048	3.190252	1.316306
Si	−3.624026	0.202153	−0.201776
O	−5.177311	0.123233	0.406373

O	−3.91736	−1.22939	−1.021111
Si	−1.737737	1.599568	−0.028376
O	−3.112793	1.513975	−1.025375
O	−2.338636	0.270332	0.812558
O	−1.746962	3.066388	0.640069
Si	−5.446379	−1.28757	−0.405692
O	−6.603343	−2.23764	−0.533195
Cl	0.344386	1.402944	2.159846

TS19

C	1.429546	−2.26586	−2.232123
C	2.545378	−2.59981	−1.491822
C	2.910004	−1.85216	−0.387523
C	2.151635	−0.75355	0.012342
C	0.971689	−0.45405	−0.686624
C	0.653603	−1.19812	−1.814129
C	3.22251	0.64412	0.548381
C	3.697372	1.197628	−0.719069
C	2.485415	1.561279	1.393382
C	3.183027	2.392879	−1.180515
C	2.075758	2.77507	0.944417
C	2.390235	3.170991	−0.359082
H	2.030273	4.118847	−0.72909
H	2.251128	1.212981	2.384629
H	1.139952	−2.83986	−3.096985
H	1.490426	3.417477	1.580852
H	−0.245039	−0.93785	−2.354003
H	3.140279	−3.46201	−1.751501
S	4.815048	0.260914	−1.612957
H	4.743118	1.003861	−2.722327
H	3.749414	−2.15896	0.212709
S	−0.194836	0.773084	−0.135551
H	−0.322243	−1.88198	1.569222
Si	−3.979904	−0.04417	0.054042
O	−5.454064	−0.72023	0.450373
O	−4.799404	1.185187	−0.729877
Si	−1.71201	−0.55604	0.450408
O	−2.911439	0.340295	1.234678
O	−2.854791	−0.91735	−0.748679
O	−1.196718	−1.8984	1.14263
Si	−6.246212	0.505718	−0.319357
O	−7.686797	0.864112	−0.549231
Cl	1.614259	−1.25754	2.180739

Cl	4.544954	−0.09474	1.459583
H	3.455124	2.755687	−2.159111

TS20

H	−2.078625	−3.8979	−1.491093
Si	0.21515	−1.72029	0.463333
O	1.282601	−2.82055	1.120958
O	1.121042	−0.49254	1.132524
Si	−1.739321	−1.76183	−0.870854
O	−0.074058	−1.83703	−1.146793
O	−1.396253	−1.72956	0.780823
O	−2.51529	−3.04982	−1.456655
Si	2.202248	−1.58581	1.732203
O	3.434396	−1.52105	2.590897
C	2.017815	0.93597	−1.365547
C	2.925664	1.434731	−0.425232
C	4.014561	0.684067	−0.023561
C	4.237198	−0.56218	−0.588189
C	3.384125	−1.0526	−1.568264
C	2.284013	−0.30563	−1.943032
Cl	2.68325	2.965531	0.321626
H	4.67133	1.061704	0.742752
H	5.080936	−1.14576	−0.253604
H	3.564553	−2.01687	−2.017066
H	1.573016	−0.69751	−2.651209
C	−1.476836	2.094281	−0.525406
C	−1.276781	3.093251	0.412681
C	−1.996103	3.062952	1.589982
C	−2.899967	2.045068	1.838904
C	−3.096308	1.060156	0.890573
C	−2.382042	1.060771	−0.310001
H	−0.555258	3.869743	0.218223
H	−1.843321	3.833869	2.32926
H	−3.459245	2.011085	2.758868
H	−1.44563	2.497021	−1.817669
H	−0.861127	2.522573	−2.587246
Cl	−4.252457	−0.16892	1.225013
S	0.453883	1.695097	−1.652709
S	−2.747303	−0.09073	−1.60611

TS21

C	−1.490465	−1.24023	1.439539

C	−0.95896	−0.35913	0.497419
C	−0.580164	0.92816	0.882563
C	−0.671634	1.284244	2.222552
C	−1.158506	0.407282	3.168584
C	−1.570156	−0.85337	2.759072
C	−3.876188	1.388753	−0.609576
C	−3.601093	0.03651	−0.776095
C	−5.107689	1.800538	−0.141279
C	−4.580677	−0.90066	−0.463184
C	−6.077645	0.860544	0.158539
C	−5.81767	−0.48726	−0.005662
H	−6.567873	−1.22678	0.221958
H	−3.114324	2.115653	−0.849913
H	−1.220602	0.700542	4.203343
H	−5.307406	2.85222	−0.01441
H	−1.783723	−2.23353	1.140775
Cl	−4.306614	−2.59337	−0.662423
H	−7.044278	1.17253	0.520909
H	−1.956897	−1.55872	3.479005
Cl	0.574457	−1.34411	−0.648278
S	−0.068147	2.131566	−0.307831
S	−1.96939	−0.35453	−1.295623
H	3.383938	3.495698	−1.088656
Si	3.478786	0.045768	−0.172646
O	5.157957	0.120392	−0.072247
O	3.592792	−1.60507	−0.007066
Si	2.009067	1.870027	−0.414774
O	2.655517	1.010724	0.876273
O	2.827967	0.829101	−1.460816
O	2.529346	3.384041	−0.676462
Si	5.239192	−1.5139	0.090361
O	6.346016	−2.5173	0.25471
H	−2.10014	−1.68264	−1.331378
H	−0.351718	2.275598	2.504393

TS22

C	3.199357	−2.34895	−1.843007
C	3.888783	−2.32961	−0.643161
C	3.342817	−1.73068	0.477279
C	2.096657	−1.13036	0.386688
C	1.365061	−1.20831	−0.79628
C	1.932712	−1.79209	−1.909298
C	2.044221	0.491493	1.304587

C	3.106103	1.283922	0.752692
C	0.725881	1.032238	1.211362
C	2.814773	2.363004	−0.051701
C	0.464072	2.138364	0.447323
C	1.503975	2.785556	−0.20831
H	1.307774	3.643862	−0.831838
H	−0.066318	0.507795	1.719665
H	3.627959	−2.81579	−2.714781
H	−0.543283	2.506315	0.341567
H	1.3477	−1.82337	−2.815375
H	4.864504	−2.78364	−0.565706
H	2.296986	0.011394	2.241366
Cl	4.117129	3.215585	−0.798724
H	3.879798	−1.71832	1.410721
H	−0.606618	−3.40036	0.4233
Si	−3.206482	−0.12893	−0.196525
O	−4.81884	−0.35726	0.167324
O	−3.526005	1.501033	−0.405192
Si	−1.259682	−1.44812	−0.329808
O	−2.04118	−0.55648	0.873
O	−2.492545	−0.98541	−1.386068
O	−1.209342	−3.04816	−0.235985
Si	−5.116994	1.252545	−0.037282
O	−6.324895	2.13799	0.074056
Cl	0.705017	−1.98949	1.938856
O	0.105575	−0.71258	−0.840345
O	4.332371	0.875719	1.004757
H	4.971644	1.40528	0.518039

TS23

C	−3.51629	2.133688	−1.897135
C	−4.283051	1.549924	−0.904438
C	−3.681584	0.727602	0.045035
C	−2.349723	0.541453	−0.111136
C	−1.488489	1.154664	−0.98937
C	−2.146971	1.950692	−1.927901
C	−2.003538	−1.53575	0.66726
C	−2.842631	−2.07503	−0.344968
C	−0.607056	−1.54452	0.470714
C	−2.302348	−2.40134	−1.570926
C	−0.083254	−1.89784	−0.746182
C	−0.935204	−2.30689	−1.764338
H	−0.524218	−2.57378	−2.725667

H	0.029322	−1.22664	1.280024
H	−3.980753	2.772023	−2.631496
H	0.981701	−1.85189	−0.906816
H	−1.517963	2.473789	−2.632304
H	−5.349348	1.704904	−0.856042
H	−4.249131	0.232748	0.816733
H	0.332548	3.438062	1.336239
Si	2.942927	0.384164	−0.013638
O	4.528203	0.437563	0.530294
O	3.32406	−1.06461	−0.793166
Si	0.884024	1.584259	0.26504
O	1.710929	0.315862	1.062368
O	2.262712	1.590907	−0.816832
O	0.958379	3.158237	0.672095
Si	4.871395	−0.97665	−0.23667
O	6.088108	−1.84878	−0.375836
Cl	−0.842791	1.272506	1.828025
Cl	−2.629796	−1.47582	2.266657
H	−2.945699	−2.75456	−2.362963
O	−0.17015	1.101767	−0.954567
O	−4.133244	−2.18509	−0.057932
H	−4.630455	−2.46996	−0.824642

TS24

H	2.057166	−4.11893	1.450581
Si	−0.640859	−1.81703	−0.287905
O	−2.126094	−2.45194	−0.533466
O	−1.061708	−0.43006	−1.050922
Si	1.448189	−2.0748	0.769892
O	−0.092457	−1.77607	1.288544
O	0.861384	−2.3735	−0.754314
O	2.328523	−3.20572	1.48923
Si	−2.641194	−0.98693	−1.265979
O	−3.571063	−0.85226	−2.456071
C	−1.278359	1.240065	1.404247
C	−1.953023	1.913007	0.356885
C	−3.099166	1.398009	−0.184554
C	−3.626027	0.168151	0.276801
C	−3.074599	−0.38381	1.455215
C	−1.935361	0.139029	1.993743
Cl	−1.29317	3.361877	−0.28734
H	−3.56854	1.894722	−1.019131
H	−4.62206	−0.11115	−0.03772

H	−3.521819	−1.26541	1.887113
H	−1.455032	−0.32302	2.838307
C	1.658482	1.582784	0.99295
C	1.786025	2.888292	0.615405
C	2.5068	3.126608	−0.542082
C	3.070246	2.084469	−1.2536
C	2.957021	0.789637	−0.78657
C	2.257952	0.49307	0.385969
H	1.293249	3.668233	1.167283
H	2.597677	4.138523	−0.902623
H	3.62102	2.266696	−2.160909
H	1.248395	1.116804	2.605263
H	2.023749	1.258091	2.465825
Cl	3.757314	−0.49262	−1.594416
O	2.340435	−0.71278	0.958657
O	−0.11778	1.597916	1.856504

TS25

C	2.491512	0.74687	−1.81266
C	1.425252	0.92676	−0.970353
C	1.128047	2.128565	−0.360209
C	1.884078	3.228495	−0.712479
C	2.933665	3.104305	−1.608397
C	3.234625	1.868448	−2.15104
C	3.05861	−0.21585	1.698897
C	2.379984	−0.91367	0.722309
C	4.229424	−0.73977	2.211901
C	2.857739	−2.12091	0.241884
C	4.70609	−1.95526	1.749303
C	4.021047	−2.64953	0.768598
H	4.382031	−3.5964	0.401552
H	2.655552	0.723878	2.041643
H	3.51575	3.972004	−1.872097
H	4.765089	−0.20073	2.976422
H	2.693612	−0.22176	−2.24015
Cl	1.984001	−2.94943	−0.985966
H	5.616131	−2.36972	2.152675
H	4.051354	1.75964	−2.84691
Cl	−0.456377	0.02105	−1.772697
H	−2.302675	2.332175	2.895186
Si	−2.792903	0.080262	0.203516
O	−4.238569	−0.32896	0.972558
O	−3.188226	−1.00608	−0.991875

Si	−1.262842	1.653217	1.041556
O	−2.579805	1.698572	0.009314
O	−1.471965	−0.00946	1.188891
O	−1.483672	2.459834	2.423348
Si	−4.601176	−1.39359	−0.223091
O	−5.729526	−2.34309	−0.512218
H	0.582476	−0.94986	−0.19614
H	1.633056	4.175046	−0.26003
O	0.149115	2.243788	0.552061
O	1.234457	−0.34598	0.216779

TS26

C	−1.436446	−1.41306	2.726288
C	−2.503822	−2.14401	2.235258
C	−2.896261	−2.01178	0.920149
C	−2.219293	−1.14091	0.072284
C	−1.084948	−0.46038	0.525776
C	−0.737471	−0.58756	1.867204
C	−3.39975	−0.27026	−1.047068
C	−4.305227	0.513601	−0.225476
C	−2.630279	0.438811	−2.023294
C	−4.142679	1.883572	−0.183203
C	−2.556091	1.804684	−2.008195
C	−3.269014	2.518303	−1.055648
H	−3.181732	3.592172	−1.002463
H	−2.065267	−0.15055	−2.725665
H	−1.127425	−1.50719	3.754439
H	−1.924498	2.327441	−2.707011
H	0.126124	−0.04253	2.21815
H	−3.036074	−2.8354	2.870219
H	−3.81779	−1.20174	−1.40955
Cl	−5.070711	2.875903	0.880148
S	−5.493261	−0.36518	0.6481
H	−5.942952	0.657298	1.379572
H	−3.699436	−2.61481	0.529455
S	−0.012985	0.465616	−0.543743
H	0.368301	−2.61864	−0.997832
Si	3.797095	−0.0203	−0.046334
O	5.318014	−0.66145	0.005709
O	4.413697	1.481518	0.265531
Si	1.623063	−0.86151	−0.42833
O	2.856738	−0.24429	−1.384535
O	2.606123	−0.62727	0.917629

O	1.226641	−2.40015	−0.600181
Si	5.966968	0.851968	0.319475
Cl	−1.650321	−2.49441	−1.716415
O	7.005124	1.395627	−0.786344
H	6.806523	1.276774	−1.710672
O	6.730163	1.033421	1.727152
H	6.267603	0.837267	2.536674

TS27

C	4.23636	−2.27134	−0.969022
C	4.637312	−1.67082	0.20835
C	3.796878	−0.82412	0.904229
C	2.49982	−0.52877	0.438113
C	2.09647	−1.16954	−0.7607
C	2.957593	−2.00871	−1.437985
C	2.044319	1.207486	0.530965
C	2.580392	1.845556	−0.67955
C	0.635806	1.478502	0.770936
C	1.762923	2.564046	−1.523757
C	−0.132051	2.232735	−0.065863
C	0.431588	2.784376	−1.210978
H	−0.172348	3.382132	−1.875835
H	0.217683	1.082402	1.679529
H	4.887624	−2.94185	−1.505058
H	−1.172139	2.395224	0.165153
H	2.603182	−2.47079	−2.346614
H	5.614061	−1.87461	0.620157
S	4.247912	1.593078	−0.996996
H	4.224332	2.14768	−2.213997
H	4.109062	−0.43438	1.857077
S	0.531625	−0.82242	−1.479288
Cl	1.212136	−1.15698	1.972739
Cl	2.922791	1.78798	1.979253
H	2.177923	2.997677	−2.420477
Si	−3.023432	−0.55872	−0.051413
O	−2.315636	−1.61406	−1.091708
O	−1.680807	−0.55717	0.901065
Si	−5.097084	0.584493	0.032263
O	−3.578489	0.902778	−0.605102
O	−4.513238	−0.88521	0.584062
Si	−0.910543	−1.62948	−0.154678
O	−6.280787	0.552525	−1.060723
H	−6.121986	0.141586	−1.905801

O	−5.622386	1.569841	1.195445
H	−5.209704	1.532087	2.05359
O	−0.618993	−3.1178	0.392694
H	0.109031	−3.20653	1.008271

TS28

H	−2.354395	−3.10463	−2.693045
Si	−0.303185	−1.71154	0.165855
O	0.544059	−2.98726	0.772634
O	0.513754	−0.67567	1.15773
Si	−1.982142	−1.27663	−1.443618
O	−0.310632	−1.45372	−1.4654
O	−1.953234	−1.68933	0.183828
O	−2.736511	−2.25541	−2.484103
Si	1.417444	−1.94881	1.769952
C	2.249422	0.927141	−1.204581
C	3.292316	1.361714	−0.379404
C	4.515889	0.714549	−0.37432
C	4.714706	−0.38418	−1.194564
C	3.689923	−0.84773	−2.005853
C	2.469234	−0.19947	−2.000359
Cl	3.095466	2.711901	0.669416
H	5.3026	1.071278	0.270489
H	5.672922	−0.88051	−1.189593
H	3.841468	−1.71057	−2.634987
H	1.651	−0.55834	−2.603778
C	−1.195201	2.186473	−0.124825
C	−0.866384	2.841151	1.048725
C	−1.655565	2.645669	2.163562
C	−2.75906	1.812205	2.11032
C	−3.080892	1.17637	0.926406
C	−2.290715	1.336171	−0.214975
H	0.007878	3.470017	1.083476
H	−1.403778	3.137685	3.090343
H	−3.379893	1.65641	2.976827
H	−1.047454	2.876558	−1.295756
H	−0.458136	2.966954	−2.034954
Cl	−4.495233	0.198912	0.887821
S	0.706439	1.780188	−1.301304
S	−2.747595	0.636798	−1.779885
O	2.999102	−1.83369	1.507076
H	3.315627	−1.42099	0.704713
O	1.260635	−2.27786	3.33996

H	0.417625	−2.57881	3.667035

TS29

C	−3.022199	−1.57324	1.199556
C	−2.059178	−1.07993	0.327612
C	−0.989212	−0.31943	0.793199
C	−0.883757	−0.10421	2.163781
C	−1.811786	−0.61362	3.048344
C	−2.881203	−1.34343	2.55256
C	−3.205278	2.278845	−0.556981
C	−3.853427	1.066099	−0.763155
C	−3.870952	3.340627	0.021027
C	−5.185021	0.93443	−0.379631
C	−5.196383	3.200242	0.392807
C	−5.854237	2.00159	0.189137
H	−6.8881	1.88372	0.469832
H	−2.173257	2.384378	−0.857164
H	−1.706145	−0.43854	4.106229
H	−3.355115	4.274429	0.175752
H	−3.839022	−2.16735	0.824953
Cl	−6.054708	−0.53554	−0.630943
H	−5.72661	4.025557	0.840793
H	−3.62038	−1.75254	3.224349
Cl	−1.48975	−2.67713	−1.056992
S	0.157655	0.42418	−0.335235
S	−2.865645	−0.22016	−1.432631
H	−3.820026	−1.14571	−1.552645
H	−0.050018	0.482174	2.519509
Si	4.0068	−0.03403	−0.070571
O	2.902988	−0.39857	1.097718
O	2.942422	−0.49508	−1.245876
Si	6.210554	0.827936	−0.088314
O	4.66615	1.480283	−0.130026
O	5.517979	−0.69743	−0.022921
Si	1.799608	−0.87414	−0.077395
O	7.102472	1.277977	1.175845
H	6.706037	1.298932	2.041987
O	7.146992	1.093763	−1.372524
H	6.87326	0.766057	−2.224422
O	1.381171	−2.40537	0.126709
H	0.539917	−2.70913	−0.241437

Table S5. Cartesian coordinates for reactants, intermediates and products involved in the formation of pre-PCTA/DT intermediates from 2-CTP on $(SiO_2)_3$ and $(SiO_2)_3O_2H_4$ clusters.

$(SiO_2)_3$

Si	−2.35427	−0.00038	−0.00011
Si	0.000008	0.00049	0.000252
O	−1.17363	0.997837	0.606269
O	−1.17283	−0.99775	−0.60608
O	−3.85336	−0.00045	−0.00021
O	1.173163	0.606713	−0.99764
O	1.173278	−0.60571	0.997844
Si	2.354278	0.000085	−0.0003
O	3.853361	−0.00099	0.00009

$(SiO_2)_3O_2H_4$

Si	0	0	0.022227
O	0.832975	−1.16901	0.834895
O	−0.82184	−1.16001	−0.82007
Si	0	2.360081	0.004789
O	−0.83298	1.169014	0.834895
O	0.821843	1.160008	−0.82007
Si	0	−2.36008	0.004789
O	0.958087	3.283842	0.910815
H	0.820637	4.22717	0.908513
O	−0.92225	3.346868	−0.88671
H	−1.61104	2.992777	−1.44258
O	−0.95809	−3.28384	0.910815
H	−0.82064	−4.22717	0.908513
O	0.922248	−3.34687	−0.88671
H	1.611036	−2.99278	−1.44258

2-CTP

C	2.406939	0.609261	0.000004
C	2.346352	−0.77453	−3E−06
C	1.12465	−1.41296	0.000007
C	−0.06932	−0.69485	0.000011
C	0.011548	0.69434	−2E−06
C	1.236224	1.340066	0.000009
H	3.356768	1.119566	0.000007
H	3.252613	−1.35941	−6E−06
H	1.082354	−2.49163	0.000017

H	1.255871	2.417955	0.000012
Cl	−1.41526	1.673835	−1.4E−05
S	−1.55187	−1.63175	−2.6E−05
H	−2.39667	−0.60171	0.000471

2-CP

C	−1.81954	−1.26813	−8.7E−05
C	−0.45027	−1.45388	−0.00022
C	0.387498	−0.35567	−9.7E−05
C	−0.11793	0.938744	0.000163
C	−1.4937	1.113351	0.0003
C	−2.33476	0.019179	0.000174
H	−2.47739	−2.12217	−0.00018
H	−0.02189	−2.4432	−0.00041
H	−1.87689	2.121266	0.000502
H	−3.40233	0.174265	0.000281
O	0.666041	2.025798	0.000284
H	1.587391	1.760664	0.000291
Cl	2.107938	−0.56816	−0.00025

IM1

Si	−2.30849	−0.43504	−0.07415
O	−3.85483	−0.68575	−0.63126
O	−2.67904	1.182045	0.134593
Si	−0.33802	−1.68087	0.323877
O	−1.71763	−1.26242	1.211018
O	−0.99519	−0.81436	−0.97461
O	0.467014	−2.96303	0.30158
Si	−4.21336	0.915205	−0.42045
O	−5.42462	1.773773	−0.63996
C	2.991615	−1.26623	−0.27845
C	2.490976	−0.07174	0.214716
C	3.147137	1.125782	−0.04388
C	4.291832	1.129813	−0.81802
C	4.788983	−0.06089	−1.31435
C	4.142728	−1.2532	−1.04442
H	2.464434	−2.1892	−0.0779
H	4.784586	2.066607	−1.02072
H	5.685096	−0.05039	−1.91431
H	4.526345	−2.18282	−1.43263
Cl	2.55573	2.622309	0.575675
H	0.488744	1.022235	0.899772

S	1.072264	−0.11869	1.290301

IM2

Si	−2.91091	0.267971	0.68438
O	−4.22744	−0.74739	0.491738
O	−3.40632	1.095497	−0.68555
Si	−0.0404	−1.12209	0.3216
O	−2.84244	1.094549	2.05649
O	−1.4888	−0.44356	0.495886
O	0.245615	−2.60526	0.290913
Si	−4.68313	0.065744	−0.87049
O	−5.81313	−0.0535	−1.85229
C	3.179386	−1.52842	−0.07458
C	2.868993	−0.17503	−0.06159
C	3.893668	0.75421	−0.21861
C	5.202273	0.341689	−0.38599
C	5.501939	−1.0063	−0.3971
C	4.489792	−1.93599	−0.24138
H	2.396801	−2.26232	0.043942
H	5.973361	1.08518	−0.50562
H	6.523753	−1.32547	−0.52751
H	4.712419	−2.99116	−0.24906
Cl	3.561831	2.446912	−0.20798
H	−3.594	1.565331	2.406075
S	1.235154	0.496539	0.148061

IM3

C	−2.30018	−0.40083	1.612258
C	−1.59949	0.576978	0.915234
C	−2.16188	1.156784	−0.2202
C	−3.41261	0.747437	−0.6489
C	−4.09363	−0.23697	0.039814
C	−3.54118	−0.8111	1.171281
H	−1.84972	−0.85529	2.480738
H	−3.8331	1.196553	−1.53348
H	−5.05549	−0.56415	−0.3202
H	−4.06554	−1.58863	1.70174
Cl	−1.34009	2.381365	−1.10244
H	0.21478	2.109823	0.845343
Si	1.356967	−0.42695	0.050539
O	0.475693	−0.54107	−1.3715
O	0.359531	−1.69564	0.65586

Si	3.693583	0.192094	−0.01835
O	2.853301	−1.20232	0.186572
O	2.282849	1.022498	−0.09888
O	5.149815	0.564486	−0.05563
Si	−0.48174	−1.73169	−0.74463
O	−1.6676	−2.49521	−1.2713
S	0.027022	0.958402	1.497713

IM4

C	1.169271	−2.67763	−0.16961
C	1.085213	−1.48854	0.54259
C	2.134329	−0.58492	0.424209
C	3.215917	−0.82103	−0.39818
C	3.276475	−2.01134	−1.09819
C	2.258658	−2.94037	−0.97844
H	0.362707	−3.38842	−0.08777
H	3.979904	−0.06918	−0.50377
H	4.116382	−2.20122	−1.74687
H	2.302185	−3.86752	−1.52724
Cl	2.080558	0.932939	1.280377
H	1.151038	4.216594	0.018819
Si	−1.3403	0.013102	0.215254
O	−2.8178	0.500543	0.835608
O	−2.16933	−0.79206	−1.00628
Si	0.879461	2.123185	−0.62815
O	−0.40776	1.176919	−0.35955
O	0.581264	3.473866	0.210707
O	1.880816	1.937084	−1.7461
Si	−3.60943	−0.3077	−0.3692
O	−5.05404	−0.52335	−0.72017
S	−0.32764	−1.19946	1.586357

IM5

C	−0.99855	1.525961	−1.76512
C	−1.607	0.888518	−0.69406
C	−1.63396	1.499248	0.555436
C	−1.05556	2.744366	0.720682
C	−0.44315	3.370191	−0.34875
C	−0.41065	2.762731	−1.59046
H	−0.98372	1.044918	−2.73098
H	−1.08763	3.211571	1.691371
H	0.01332	4.336556	−0.20528

H	0.070398	3.247596	−2.42423
Cl	−2.3997	0.751269	1.900063
H	−3.15735	−0.78841	−0.00866
Si	1.27471	−0.94945	0.041804
O	2.848674	−1.28479	0.486026
O	1.728847	0.637661	−0.22114
Si	−0.72731	−2.2032	−0.12756
O	0.557022	−1.79568	−1.16195
O	0.042495	−1.27779	1.065846
O	−1.52669	−3.47914	0.009725
Si	3.279409	0.285578	0.228171
O	4.542712	1.088887	0.347148
S	−2.26793	−0.7215	−1.00555

IM6

C	1.387429	−1.10029	−1.81333
C	1.831493	−0.57752	−0.60455
C	1.818532	−1.3877	0.527558
C	1.354171	−2.68969	0.44654
C	0.912591	−3.19345	−0.76123
C	0.9314	−2.4007	−1.89545
H	1.401245	−0.46753	−2.68648
H	1.345748	−3.29812	1.336149
H	0.550041	−4.20801	−0.81129
H	0.587133	−2.78955	−2.84037
Cl	2.388544	−0.815	2.045534
H	1.448736	4.033568	0.266961
Si	−1.40171	0.752646	0.026493
O	−3.01528	1.049489	0.324116
O	−1.75307	−0.87623	−0.06569
Si	0.595136	1.980378	−0.19417
O	−0.64385	1.496957	−1.22534
O	−0.25147	1.259111	1.075446
O	0.703143	3.586514	−0.12333
Si	−3.3467	−0.56693	0.232814
O	−4.5825	−1.40969	0.364995
S	2.446128	1.094137	−0.57186

IM7

Si	−1.67396	0.674086	−0.10713
O	−1.10323	1.955799	0.751603
O	−0.54335	0.930352	−1.27869

Si	−3.44718	−0.87645	0.134507
O	−1.83235	−0.82902	0.57211
O	−3.26959	0.637138	−0.54666
Si	0.065275	2.233872	−0.42356
O	−4.43024	−0.97015	1.405922
H	−5.2007	−1.52899	1.350676
O	−3.8828	−2.037	−0.90746
H	−3.52045	−2.04741	−1.78939
O	1.601759	2.019628	−0.0113
H	2.185807	2.770187	0.050994
O	−0.06768	3.693201	−1.11346
H	−0.93109	4.065566	−1.27068
C	1.148183	−2.03344	−0.36885
C	1.892981	−1.19512	0.454941
C	3.147364	−0.79232	0.009816
C	3.638279	−1.19365	−1.21813
C	2.879762	−2.01698	−2.0278
C	1.633956	−2.43823	−1.59551
H	0.175663	−2.36442	−0.03995
H	4.615773	−0.85897	−1.52607
H	3.264434	−2.32897	−2.98574
H	1.035394	−3.0877	−2.21525
Cl	4.13496	0.209632	1.013346
H	0.148873	−1.31704	1.94228
S	1.305676	−0.65552	2.018722

IM8

Si	2.064299	0.175674	0.057371
O	1.12211	1.489073	0.206458
O	1.131338	−1.15314	0.136297
Si	4.41327	0.153035	−0.13653
O	3.134606	0.130916	−1.20854
O	3.325041	0.19028	1.128149
Si	−0.47407	−1.22922	0.459867
O	5.361019	1.454279	−0.237
H	6.298592	1.318849	−0.34329
O	5.388825	−1.1412	−0.19241
H	5.011192	−2.01644	−0.17781
O	−0.9582	−2.75215	0.731567
H	−1.06249	−3.02341	1.640729
O	−0.8601	−0.36312	1.783008
H	−0.61742	0.562986	1.800038
C	−3.95252	−1.16388	−0.82975

C	−2.98706	−0.17686	−0.67529
C	−3.35568	1.017303	−0.0648
C	−4.65053	1.220973	0.377835
C	−5.59581	0.226666	0.215224
C	−5.24719	−0.96871	−0.38853
H	−3.66632	−2.09163	−1.29919
H	−4.90613	2.156941	0.847229
H	−6.60402	0.390489	0.561768
H	−5.98004	−1.74939	−0.51735
Cl	−2.21629	2.296824	0.170367
H	0.40261	1.626255	−0.41208
S	−1.35453	−0.46162	−1.30717

IM9

Si	−2.09003	0.533715	−0.11083
O	−1.12858	1.038787	−1.34953
O	−1.51499	1.745739	0.849802
Si	−3.67246	−1.19775	0.196766
O	−3.7237	0.389354	−0.31908
O	−2.02143	−1.0386	0.405773
Si	−0.56133	2.304802	−0.40744
O	−4.05938	−2.30727	−0.90574
H	−4.80212	−2.87973	−0.73393
O	−4.54672	−1.50946	1.523578
H	−4.53553	−0.90059	2.257242
O	−1.01248	3.76565	−0.92411
H	−0.32977	4.399099	−1.12757
O	1.034638	2.360696	−0.17003
H	1.502791	1.568328	0.089704
C	3.317867	0.238691	1.59975
C	2.728426	−0.61533	0.66826
C	3.513542	−1.06297	−0.39097
C	4.833672	−0.66531	−0.51625
C	5.398063	0.181318	0.417469
C	4.63484	0.632273	1.480539
H	2.724388	0.592979	2.429928
H	5.409216	−1.02909	−1.35207
H	6.426975	0.485403	0.310819
H	5.061055	1.294409	2.217383
Cl	2.869568	−2.12762	−1.58841
H	0.820093	−1.6589	−0.20452
S	1.043521	−1.03028	0.948684

IM10

Si	0.654882	0.652954	0.381768
O	−0.14552	1.907433	1.116944
O	−0.14576	0.985964	−1.03759
Si	3.467426	−0.31377	0.026983
O	2.257317	0.754359	0.323213
O	3.016008	−1.34664	−1.152
Si	−0.96419	2.239789	−0.3028
O	3.726232	−1.02313	1.46501
H	4.461115	−1.62236	1.558039
O	4.817044	0.429393	−0.47183
H	4.880858	0.68978	−1.38648
O	−0.64655	3.70163	−0.90916
H	−1.38383	4.246239	−1.17077
O	−2.5755	2.088121	−0.25909
H	−2.96939	1.268403	0.039792
C	−2.43702	−0.71664	1.694065
C	−1.53772	−1.16506	0.733359
C	−2.02625	−1.54516	−0.51537
C	−3.38002	−1.46102	−0.79565
C	−4.26112	−1.01101	0.171189
C	−3.79183	−0.64147	1.421547
H	−2.05318	−0.41609	2.656023
H	−3.7323	−1.7491	−1.77263
H	−5.31377	−0.95072	−0.05644
H	−4.47323	−0.29032	2.180097
Cl	−0.97686	−2.12323	−1.74694
H	2.193462	−1.82579	−1.06245
S	0.185045	−1.25598	1.148173

IM11

Si	1.713027	0.565079	0.07458
O	2.306643	−0.30946	−1.19362
O	2.268851	−0.57677	1.133222
Si	0.682194	2.674394	0.3328
O	2.253972	2.118602	0.277238
O	0.155264	1.098471	0.135641
Si	2.911036	−1.45586	−0.13376
O	0.317647	3.680035	−0.8734
H	−0.21733	4.44433	−0.67691
O	0.205999	3.383527	1.708706
H	0.250507	2.906239	2.532872

O	4.5254	−1.59771	−0.16936
H	5.071045	−0.81765	−0.22214
O	2.321059	−2.9521	−0.24722
H	2.933723	−3.65468	−0.44932
C	−2.19291	−0.21142	−1.74862
C	−2.13971	−0.99194	−0.59688
C	−3.16634	−0.85047	0.329238
C	−4.20666	0.037917	0.114943
C	−4.24342	0.797629	−1.03695
C	−3.2301	0.667803	−1.97273
H	−1.39209	−0.29381	−2.46769
H	−4.98316	0.120728	0.858285
H	−5.05861	1.484882	−1.1991
H	−3.24121	1.258917	−2.87496
Cl	−3.18291	−1.77426	1.793116
H	−0.94309	−2.41238	0.820589
S	−0.77261	−2.08117	−0.45869

IM12

Si	−1.12158	0.872603	−0.00796
O	−0.26216	1.555127	−1.23868
O	0.041065	1.32384	1.072514
Si	−3.17381	−0.27704	0.171329
O	−2.68866	1.322485	0.25784
O	−1.58162	−0.71203	−0.08129
Si	0.9803	1.947848	−0.17351
O	−4.14883	−0.61781	−1.06568
H	−4.99012	−1.02712	−0.88268
O	−3.8727	−0.86345	1.509112
H	−3.45928	−0.74102	2.359446
O	1.351077	3.515937	−0.06736
H	0.67142	4.167413	0.08593
C	1.552672	−1.1373	−1.82944
C	2.021746	−0.66666	−0.60864
C	1.927313	−1.49175	0.508949
C	1.371455	−2.75539	0.40175
C	0.909206	−3.20589	−0.81886
C	0.996474	−2.39646	−1.93728
H	1.621573	−0.49231	−2.69082
H	1.305544	−3.37452	1.281533
H	0.473286	−4.18994	−0.89092
H	0.629577	−2.73972	−2.89133
Cl	2.494945	−0.97813	2.049618

S	2.772912	0.946596	−0.52895

IM13

C	1.58605	2.681615	−0.41455
C	0.719455	2.075263	0.480437
C	−0.58884	2.532811	0.596294
C	−1.0315	3.570734	−0.20156
C	−0.16549	4.170283	−1.09592
C	1.140626	3.728629	−1.19917
H	2.595835	2.316329	−0.50822
H	−2.05657	3.892808	−0.11544
H	−0.51845	4.980704	−1.71363
H	1.82012	4.189762	−1.89759
Cl	−1.70278	1.810421	1.695288
Si	2.284981	−0.8297	0.00736
O	3.524006	−1.81939	0.585817
O	3.545609	0.371438	−0.16729
Si	0.338185	−1.68496	−1.09874
O	1.667117	−0.79503	−1.54583
O	0.109229	−3.12204	−1.80432
Si	4.6796	−0.67448	0.354179
O	6.166846	−0.61958	0.580678
S	1.325696	0.75776	1.511574
H	−0.51786	−3.19944	−2.51898
C	−3.76649	−0.51763	−0.10797
C	−4.7543	−1.04293	0.704691
C	−4.53011	−2.22147	1.390388
C	−3.31455	−2.8674	1.258491
C	−2.32851	−2.33912	0.44754
C	−2.53601	−1.15529	−0.2529
H	−5.69322	−0.52028	0.790495
H	−5.30277	−2.62879	2.022706
H	−3.12372	−3.78702	1.788546
H	−1.38858	−2.86429	0.375835
S	−1.32402	−0.38838	−1.2896
Cl	−4.08785	0.952733	−0.95236
O	0.939519	−1.81997	0.424534
H	0.159615	0.12804	1.692221

IM14

C	0.687277	2.873358	−1.78707
C	1.017183	2.571801	−0.47145

C	0.153057	2.969056	0.544855
C	−1.01233	3.654909	0.245203
C	−1.32187	3.952389	−1.06793
C	−0.47338	3.559202	−2.08785
H	1.358728	2.561035	−2.57103
H	−1.66468	3.957417	1.04865
H	−2.23059	4.48939	−1.2904
H	−0.71211	3.78381	−3.11517
Cl	0.499824	2.631928	2.197077
Si	1.907506	−0.19061	−0.26438
O	3.213279	−1.10526	0.011812
Si	−0.24431	−1.05885	−0.72998
O	1.07966	−0.61146	−1.64655
O	0.585796	−0.61099	0.649959
O	−0.5887	−2.63746	−0.69588
Si	4.464637	−2.05057	0.297671
O	5.374569	−2.7192	−0.70276
S	2.572255	1.781599	−0.13076
H	−1.3759	−2.95811	−1.13415
C	−3.76766	−1.55516	0.05288
C	−4.53333	−1.96387	1.129865
C	−4.41257	−1.31168	2.342597
C	−3.52493	−0.25909	2.480273
C	−2.76671	0.14681	1.399337
C	−2.88097	−0.48956	0.169346
H	−5.21214	−2.79261	1.010894
H	−5.00981	−1.63546	3.1804
H	−3.417	0.243973	3.427927
H	−2.06369	0.960021	1.494095
S	−1.91956	0.112163	−1.20331
Cl	−3.92135	−2.41883	−1.43657
O	4.58254	−2.13434	1.905977
H	5.300491	−2.67143	2.238097

IM15

C	−1.91603	1.940358	1.656846
C	−1.65581	2.086593	0.298556
C	−0.52272	2.789609	−0.09963
C	0.326602	3.338115	0.845765
C	0.06518	3.170381	2.19113
C	−1.05426	2.466869	2.599749
H	−2.80231	1.40729	1.966403
H	1.194718	3.884129	0.515143

H	0.739564	3.592579	2.919349
H	−1.26522	2.33411	3.648863
Cl	−0.14212	2.995826	−1.76352
Si	−2.56889	−0.5509	−0.34717
O	−3.64235	−1.52458	−1.19956
O	−3.3734	−1.00596	1.070371
Si	0.51805	−1.43372	−0.50152
O	−1.06089	−1.06266	−0.32325
O	0.711796	−3.05401	−0.45675
Si	−4.41133	−1.93831	0.198352
O	−5.57779	−2.82418	0.536834
S	−2.80351	1.439313	−0.90251
H	0.426402	−3.54005	0.311845
C	3.945268	−0.08001	−0.11117
C	5.245469	−0.46484	−0.39413
C	5.736436	−1.65991	0.094586
C	4.931433	−2.47333	0.873113
C	3.635975	−2.08512	1.153914
C	3.120503	−0.89146	0.663777
H	5.862322	0.180637	−0.9981
H	6.750349	−1.95055	−0.13167
H	5.308939	−3.40559	1.262346
H	3.001412	−2.70758	1.764711
S	1.458728	−0.41809	1.082872
Cl	3.387261	1.420447	−0.74098
O	1.082425	−0.9625	−1.9445
H	1.336652	−1.64255	−2.56385

IM16

C	1.251748	1.438769	0.115942
C	−0.04692	1.942226	0.194803
C	−0.39058	3.047598	−0.58414
C	0.532206	3.646251	−1.42007
C	1.814784	3.13967	−1.49223
C	2.167817	2.047251	−0.72848
H	0.234078	4.497351	−2.00988
H	2.536988	3.601148	−2.147
H	3.169733	1.647438	−0.7796
Cl	−1.98063	3.709111	−0.54495
S	−1.26979	1.23843	1.285862
C	3.868304	−0.94636	0.64129
C	4.426522	−0.35632	1.786072
C	3.654601	0.534144	2.553605

C	2.386341	0.837869	2.201368
C	1.729444	0.281972	0.980971
C	2.596851	−0.67873	0.221516
H	5.436035	−0.60553	2.064408
H	4.087548	0.972907	3.439346
H	1.788124	1.511852	2.795634
H	0.852633	−0.27156	1.322012
S	2.034357	−1.39782	−1.28241
Cl	4.880788	−2.04125	−0.23269
Si	−1.63975	−0.49772	0.17244
O	−3.18643	−0.90777	0.373546
Si	−0.04549	−1.67638	−1.12241
O	−0.62216	−1.79568	0.440855
O	−1.08958	−0.40034	−1.38738
O	−0.40386	−2.87151	−2.15097
Si	−4.68847	−1.35213	0.682336
O	−5.29036	−1.68818	2.023728
H	0.197961	−3.60175	−2.26963
O	−5.43237	−1.35985	−0.74854
H	−6.35578	−1.60767	−0.73767

IM17

C	2.505005	0.722942	−0.18421
C	2.135041	1.827514	0.537129
C	2.333154	3.125162	0.171948
C	2.993902	3.362896	−1.024
C	3.409983	2.283274	−1.78667
C	3.171202	0.981501	−1.38256
H	3.175901	4.377132	−1.34364
H	3.930081	2.463615	−2.71409
H	3.50818	0.156247	−1.99023
Cl	1.78715	4.429958	1.16099
S	−0.94724	−0.93124	2.651403
C	1.421974	−2.88387	0.244974
C	1.938393	−3.15155	1.500838
C	2.61845	−2.1637	2.182692
C	2.774848	−0.91584	1.613436
C	2.271896	−0.63671	0.349533
C	1.579747	−1.63168	−0.35325
H	1.800945	−4.13171	1.927091
H	3.022288	−2.36833	3.161697
H	3.304584	−0.13793	2.140241
H	−1.5832	0.040413	3.313728

S	0.958478	−1.33844	−1.99695
Cl	0.588376	−4.15508	−0.56133
Si	−1.67596	−0.30191	0.80742
O	−3.12675	0.462972	1.074761
Si	−0.87113	−0.50872	−1.41913
O	−1.76487	−1.47041	−0.35042
O	−0.74968	0.64098	−0.18862
O	−1.63404	−0.04375	−2.74927
Si	−4.07546	1.08988	−0.05683
O	−3.85881	1.006434	−1.55505
H	−2.49834	0.373378	−2.58023
O	−5.29954	1.805178	0.69497
H	−5.96067	2.228963	0.149419

IM18

C	1.279665	1.60481	−0.04888
C	0.037403	2.230274	−0.07858
C	−0.19712	3.288949	−0.94683
C	0.801933	3.737608	−1.78694
C	2.042183	3.122163	−1.76715
C	2.270378	2.069767	−0.90468
H	0.608148	4.558663	−2.45918
H	2.828966	3.460397	−2.42319
H	3.235932	1.584645	−0.88469
S	−1.30902	1.767793	1.005796
C	3.608546	−1.00397	0.751899
C	4.205825	−0.39197	1.864881
C	3.533497	0.651533	2.525923
C	2.320643	1.073411	2.106125
C	1.622734	0.488836	0.922691
C	2.389686	−0.62324	0.267958
H	5.169757	−0.7367	2.197576
H	3.998751	1.114472	3.382569
H	1.7995	1.8688	2.616999
H	0.68496	0.066315	1.290634
S	1.79132	−1.38172	−1.20293
Cl	4.508547	−2.27636	0.002651
H	−1.17141	3.750885	−0.96086
Si	−1.80717	−0.03779	0.073468
O	−3.38485	−0.29273	0.302511
Si	−0.30644	−1.47384	−1.06382
O	−0.90689	−1.38267	0.493064
O	−1.23866	−0.15142	−1.4777

O	−0.75499	−2.7338	−1.97187
Si	−4.95242	−0.54772	0.456659
O	−6.01446	−0.516	−0.61316
H	−0.20807	−3.51373	−2.01646
O	−5.17507	−0.83394	2.030058
H	−6.07732	−1.00648	2.295648

IM19

C	1.698227	1.226587	−0.08578
C	0.48441	1.910268	−0.02675
C	0.25031	2.968953	−0.90645
C	1.175823	3.356785	−1.84699
C	2.372252	2.667711	−1.93406
C	2.613639	1.630173	−1.06447
H	0.957214	4.176911	−2.51245
H	3.113569	2.934738	−2.67056
H	3.552603	1.098234	−1.12621
S	−0.90182	1.600569	1.068229
C	3.827185	−1.76395	0.214548
C	4.805406	−1.26015	1.069913
C	4.625447	−0.00211	1.67421
C	3.470065	0.677353	1.508531
C	2.29668	0.118876	0.776488
C	2.652534	−1.09569	−0.01936
H	5.714559	−1.81725	1.227107
H	5.415993	0.427799	2.26907
H	3.308418	1.638694	1.970135
S	1.733883	−1.70219	−1.39441
H	−0.69799	3.477667	−0.84216
H	4.01256	−2.68426	−0.31785
Cl	1.241606	−0.48387	2.170358
Si	−1.66821	−0.03826	0.026704
O	−3.27072	−0.04875	0.238877
Si	−0.36651	−1.56102	−1.22024
O	−1.01495	−1.54702	0.309045
O	−1.10806	−0.10269	−1.53642
O	−0.9072	−2.6838	−2.25203
Si	−4.85315	−0.00706	0.424063
O	−5.90546	0.348788	−0.59626
H	−0.45729	−3.51961	−2.33947
O	−5.1084	−0.41291	1.966269
H	−6.02344	−0.43037	2.242989

IM20

C	−2.18362	1.934999	1.974051
C	−2.33294	1.947787	0.615911
C	−1.51306	2.842783	−0.07877
C	−0.62821	3.654308	0.612411
C	−0.53482	3.58306	1.991194
C	−1.32842	2.698761	2.713561
H	−0.00333	4.333885	0.057095
H	0.166807	4.219892	2.508121
H	−1.26659	2.626764	3.787462
Cl	−1.58393	2.952846	−1.79019
Si	−2.40252	−0.75972	−0.3523
O	−3.2346	−1.95206	−1.19449
O	−2.36152	−1.82223	0.975897
Si	0.606942	−1.02457	−0.52074
O	−0.90462	−0.43414	−0.82115
O	0.463942	−2.39996	0.350717
Si	−3.1884	−2.95561	0.111381
O	−3.69	−4.34129	0.404559
S	−3.57385	0.946992	−0.17571
H	−0.10371	−2.38477	1.121275
C	4.181083	0.066463	−0.34844
C	5.424173	−0.5309	−0.22224
C	5.668676	−1.40786	0.817136
C	4.673635	−1.68895	1.736689
C	3.435628	−1.08944	1.609324
C	3.168428	−0.21203	0.565864
H	6.190678	−0.30144	−0.94453
H	6.640069	−1.86858	0.905884
H	4.858232	−2.37092	2.55145
H	2.653233	−1.29891	2.321292
S	1.570908	0.562331	0.457781
Cl	3.924111	1.150379	−1.65832
O	1.384229	−1.41923	−1.88389
H	1.461282	−2.34399	−2.10644

IM21

C	0.939403	1.506839	0.049364
C	−0.38048	1.945316	0.174303
C	−0.81777	3.026917	−0.5903
C	0.04024	3.67534	−1.45828
C	1.34718	3.244541	−1.56668

C	1.789251	2.171981	−0.81931
H	−0.32521	4.506314	−2.03866
H	2.021527	3.747811	−2.24142
H	2.81033	1.833882	−0.90842
Cl	−2.44156	3.590542	−0.48984
Si	−2.12815	−0.39876	0.183645
O	−3.69832	−0.33621	−0.40681
O	−2.60381	−1.72576	1.126918
Si	0.129663	−1.97689	−0.94474
O	−1.04451	−0.82116	−0.92287
O	0.169574	−2.67807	0.524024
Si	−4.13335	−1.63587	0.502128
O	−5.38993	−2.43312	0.705664
S	−1.48311	1.195689	1.36105
H	−0.65399	−2.88614	0.968823
C	3.800051	−0.52007	0.586633
C	4.225818	0.113318	1.765375
C	3.295236	0.814992	2.552333
C	2.002374	0.923295	2.175219
C	1.492443	0.36178	0.889155
C	2.511123	−0.45178	0.145008
H	5.256531	0.0269	2.063762
H	3.625882	1.264873	3.475805
H	1.28524	1.45111	2.785681
H	0.657344	−0.30158	1.133483
S	2.043238	−1.20966	−1.37003
Cl	4.991348	−1.4117	−0.28993
O	−0.28928	−2.93928	−2.18215
H	0.240377	−3.70478	−2.38898

IM22

C	1.513019	1.915823	−0.25573
C	0.445149	2.609108	0.252783
C	−0.18756	3.662073	−0.33691
C	0.306841	4.100904	−1.55656
C	1.400772	3.455134	−2.11015
C	1.999208	2.378026	−1.48005
H	−0.16078	4.935715	−2.0549
H	1.792726	3.800449	−3.05369
H	2.850664	1.891832	−1.92953
Cl	−1.54505	4.422929	0.402391
Si	−2.00235	−0.43878	0.494512
O	−3.5425	−0.13116	−0.09137

O	−2.47312	−2.06953	0.666596
Si	0.099096	−1.56624	−1.28457
O	−0.78732	−0.38201	−0.55408
O	0.212342	−2.83318	−0.2719
Si	−3.96911	−1.71371	0.070949
O	−5.21275	−2.51408	−0.19296
S	−1.63072	0.633647	2.231059
H	−0.59163	−3.12866	0.159672
C	3.041079	−1.38878	0.814304
C	3.306017	−1.11898	2.14594
C	2.965106	0.109179	2.673693
C	2.384214	1.068231	1.867945
C	2.120377	0.816494	0.524733
C	2.427167	−0.44282	−0.00865
H	3.775998	−1.87576	2.752159
H	3.168406	0.323263	3.711207
H	2.147347	2.04389	2.26398
H	−0.30408	0.47309	2.124295
S	2.026296	−0.84607	−1.6968
Cl	3.498549	−2.93144	0.212161
O	−0.67915	−1.90011	−2.67442
H	−0.47259	−2.70394	−3.14427

IM23

C	0.751794	1.643445	−0.12048
C	−0.57441	2.018766	0.078438
C	−1.1952	2.915874	−0.78292
C	−0.497	3.461696	−1.84109
C	0.829751	3.116466	−2.03416
C	1.439283	2.218235	−1.18189
H	−0.98761	4.152618	−2.50827
H	1.388396	3.540664	−2.85383
H	2.470674	1.93793	−1.33932
Si	−2.31198	−0.17807	0.403169
O	−3.39864	0.24476	−0.8092
O	−3.5849	−0.91862	1.21803
Si	0.117738	−2.14811	−0.27974
O	−1.19472	−1.19855	−0.09767
O	0.655026	−2.58751	1.194153
Si	−4.62635	−0.47802	0.017134
O	−6.10159	−0.64996	−0.2125
S	−1.52917	1.42337	1.479207
H	0.042443	−2.95658	1.826531

C	3.744315	−0.23149	0.295451
C	4.321871	0.580199	1.286718
C	3.500266	1.405103	2.075193
C	2.163771	1.452926	1.879829
C	1.481722	0.67842	0.801757
C	2.404531	−0.23208	0.043151
H	5.386417	0.540788	1.441889
H	3.952481	2.003099	2.851479
H	1.535316	2.081052	2.493272
H	0.719043	0.057717	1.28449
S	1.723855	−1.20292	−1.25768
Cl	4.820768	−1.24437	−0.59797
O	−0.40961	−3.35957	−1.22437
H	0.193403	−4.05505	−1.47383
H	−2.22916	3.173958	−0.61673

IM24

C	−1.26084	1.419224	0.160849
C	0.011324	1.932722	−0.09288
C	0.55356	2.888782	0.769157
C	−0.13876	3.363585	1.859814
C	−1.41359	2.886277	2.101769
C	−1.94859	1.935742	1.263401
H	0.313471	4.097344	2.507863
H	−1.98766	3.24243	2.942461
H	−2.94163	1.560447	1.464442
Si	2.083418	0.044462	−0.43105
O	3.163093	0.621397	0.731095
O	3.404475	−0.5333	−1.29484
Si	0.077104	−2.21566	0.657672
O	1.119574	−1.07703	0.156552
O	−0.27812	−3.29416	−0.5013
Si	4.432696	0.047973	−0.14361
O	5.927237	0.065831	0.018948
S	1.072805	1.527296	−1.48425
H	−0.79728	−2.99806	−1.24658
C	−3.97422	−1.01463	0.285943
C	−4.89802	−0.3545	−0.52629
C	−4.49132	0.742605	−1.30628
C	−3.1965	1.135192	−1.32516
C	−2.13478	0.413557	−0.58421
C	−2.65701	−0.64606	0.327322
H	−5.92795	−0.67245	−0.53136

H	−5.21932	1.286006	−1.88814
H	−2.87031	1.97624	−1.91655
S	−1.63806	−1.34836	1.57615
O	0.816509	−2.99667	1.872142
H	0.550476	−3.89208	2.063877
H	1.547583	3.250745	0.560036
Cl	−1.26797	−0.56893	−1.93965
H	−4.30268	−1.82024	0.924022

IM25

C	0.955878	3.164212	−0.85689
C	0.429005	3.59557	0.34524
C	−0.51213	2.822036	1.003229
C	−0.93622	1.604344	0.492641
C	−0.38804	1.17911	−0.71746
C	0.535663	1.96014	−1.39254
C	−1.94198	0.776677	1.277397
C	−3.19112	0.453235	0.498032
C	−1.30148	−0.4232	1.891315
C	−3.70513	−0.8129	0.510203
C	−1.86165	−1.65383	1.842879
C	−3.07456	−1.87817	1.16559
H	−3.53519	−2.85165	1.149208
H	−0.36296	−0.26487	2.397638
H	1.689594	3.757483	−1.37901
H	−1.37408	−2.48144	2.33743
H	0.931243	1.611697	−2.33434
H	0.74412	4.534582	0.772721
H	−2.25864	1.432454	2.101074
Cl	−5.16965	−1.18573	−0.33712
S	−3.85961	1.832867	−0.31895
H	−4.99279	1.250187	−0.71295
H	−0.93032	3.16844	1.937419
S	−0.88188	−0.36961	−1.45324
H	−0.64232	−3.27862	−0.38826
Si	2.849042	−0.74767	−0.13337
O	4.239562	−1.41981	0.499842
O	3.635291	0.724105	−0.19962
Si	0.687031	−1.51653	−0.69389
O	2.171072	−1.36794	−1.48751
O	1.435765	−0.88536	0.68597
O	0.263525	−3.07318	−0.61286
Si	5.001678	0.042985	0.429462

O	6.367439	0.552275	0.791229

IM26

C	1.222811	3.593283	−0.01416
C	2.505426	3.476057	−0.50973
C	3.110297	2.23436	−0.58472
C	2.457271	1.079922	−0.17763
C	1.151825	1.210019	0.318187
C	0.555548	2.459816	0.408308
C	3.074392	−0.30134	−0.33634
C	3.063086	−1.1381	0.888118
C	2.546503	−0.99053	−1.52913
C	2.817128	−2.48949	0.817139
C	2.279999	−2.31847	−1.53837
C	2.429822	−3.09052	−0.36923
H	2.226322	−4.1486	−0.39189
H	2.490917	−0.3933	−2.42819
H	0.741556	4.556146	0.053107
H	1.958306	−2.79431	−2.45273
H	−0.44178	2.535792	0.812518
H	3.047093	4.349306	−0.83763
S	3.455353	−0.30143	2.359345
H	3.388012	−1.37562	3.14802
H	4.113237	2.158899	−0.96541
S	0.212346	−0.18807	0.929286
H	0.272552	−0.65989	−2.12327
Si	−3.54438	−0.14624	−0.13826
O	−4.94362	−0.28151	−1.03494
O	−4.4803	0.046275	1.229217
Si	−1.2319	−0.20702	−0.57772
O	−2.42009	−1.33912	−0.1904
O	−2.42269	0.986401	−0.52165
O	−0.61469	−0.30101	−2.0633
Si	−5.85756	−0.09118	0.32757
O	−7.32917	−0.05531	0.624086
Cl	4.935761	−0.1108	−0.72279
H	2.886242	−3.08648	1.713895

IM27

C	1.152125	2.869055	−1.23156
C	0.693357	2.385781	−0.0167
C	−0.14307	1.273443	0.042926

C	−0.52741	0.656756	−1.15728
C	−0.06518	1.149286	−2.3671
C	0.774044	2.24834	−2.40266
C	−3.42042	1.166771	−0.66784
C	−3.09868	−0.18418	−0.71248
C	−4.68516	1.581949	−0.29593
C	−4.08994	−1.10422	−0.38498
C	−5.65787	0.654104	0.031235
C	−5.35745	−0.69371	−0.01783
H	−6.09744	−1.43778	0.229012
H	−2.67574	1.900585	−0.93261
H	−0.37338	0.662531	−3.27816
H	−4.90778	2.637115	−0.26779
H	1.80421	3.726899	−1.24178
Cl	−3.74839	−2.7956	−0.44033
H	−6.64642	0.973283	0.320542
H	1.13506	2.621719	−3.34778
Cl	1.198063	3.19512	1.412576
S	−0.75934	0.675476	1.601192
S	−1.51791	−0.80544	−1.18705
H	0.291321	−1.07691	4.059113
Si	2.624864	−0.89376	0.390444
O	4.040109	−1.77134	0.300574
O	2.982178	−0.23202	−1.09967
Si	0.838933	−0.63339	1.900731
O	1.189774	−1.65497	0.612579
O	2.341819	0.101976	1.65884
O	0.691453	−1.45237	3.280039
Si	4.381646	−1.105	−1.17131
O	5.502071	−1.22753	−2.16387

IM28

C	−0.96323	−0.93458	2.910741
C	−0.31943	−1.43785	1.792143
C	−1.05238	−2.094	0.813451
C	−2.41993	−2.27185	0.978087
C	−3.05411	−1.78648	2.106802
C	−2.32235	−1.12106	3.076974
C	2.803401	1.452513	1.877586
C	2.713404	0.339451	1.058486
C	3.575127	2.538656	1.508613
C	3.372121	0.34322	−0.15781
C	4.243079	2.525192	0.295426

C	4.145004	1.425517	−0.53894
H	4.667039	1.397519	−1.48235
H	2.269101	1.467767	2.818589
H	−4.11767	−1.92167	2.223696
H	3.650387	3.394163	2.161864
H	−0.39893	−0.39736	3.661799
Cl	3.282604	−1.02996	−1.20779
H	4.84238	3.369634	−0.00654
H	−2.81336	−0.73554	3.957364
S	−0.2436	−2.7913	−0.62453
S	1.524897	−1.07295	1.515078
H	0.190007	−0.75365	−3.91947
Si	−1.63503	0.837532	−1.05187
O	−2.08262	2.318521	−1.68288
O	−2.26456	1.31475	0.417623
Si	−0.55321	−1.11934	−1.81641
O	−2.14674	−0.53949	−1.77302
O	−0.07236	0.360807	−1.15308
O	0.039444	−1.44386	−3.27862
Si	−2.6946	2.774899	−0.21873
O	−3.36603	4.007379	0.316416
H	1.56279	−0.6167	2.773807
H	−2.97851	−2.78722	0.212084

IM29

C	1.238728	1.282839	1.126282
C	1.94023	0.268034	0.500717
C	3.053254	0.570423	−0.27025
C	3.457934	1.883757	−0.41717
C	2.748983	2.898315	0.200306
C	1.639545	2.597201	0.969697
H	0.389038	1.032345	1.743068
H	4.324503	2.099712	−1.02084
H	3.066751	3.921278	0.077728
H	1.082518	3.382692	1.454801
Cl	3.921545	−0.69708	−1.04448
H	1.109121	−3.68888	0.356397
Si	−1.83485	−0.43994	−0.02667
O	−3.4314	−0.75058	−0.37954
O	−2.182	1.190638	−0.0272
Si	0.122258	−1.6832	0.359642
O	−1.12929	−1.09225	1.306283
O	−0.6408	−1.02831	−0.98407

O	0.249315	−3.27926	0.41286
Si	−3.76607	0.869423	−0.37546
O	−4.99622	1.702614	−0.58828
O	1.579261	−1.03638	0.645244

IM30

C	2.900389	−3.39206	−1.11425
C	4.140608	−2.84461	−0.83863
C	4.226281	−1.54569	−0.36942
C	3.092082	−0.77768	−0.15991
C	1.858447	−1.34884	−0.44445
C	1.754437	−2.64138	−0.92286
C	3.196499	0.627998	0.400995
C	2.50621	1.632811	−0.45968
C	2.731923	0.685047	1.821719
C	1.531425	2.450425	0.018958
C	1.744693	1.520208	2.237271
C	1.104112	2.405687	1.352702
H	0.308283	3.050537	1.682035
H	3.245465	0.040927	2.520826
H	2.820057	−4.40161	−1.48556
H	1.44734	1.524993	3.276051
H	0.778429	−3.04339	−1.14875
H	5.037925	−3.4223	−0.99402
H	4.265268	0.873747	0.368945
Cl	0.783276	3.561339	−1.08027
H	5.193612	−1.11092	−0.16307
H	0.855914	−0.97398	2.498031
Si	−2.72396	−0.46656	0.137534
O	−4.1874	−0.50016	0.936432
O	−3.53815	−0.10054	−1.27031
Si	−0.48281	−0.79738	0.751537
O	−1.52436	0.513822	0.67898
O	−1.73179	−1.77115	0.192576
O	0.003602	−1.29357	2.201521
Si	−4.98176	−0.14071	−0.46658
O	−6.41823	0.061781	−0.85398
O	0.735078	−0.58468	−0.29188
O	2.937524	1.63431	−1.71909
H	2.438511	2.274284	−2.23348

IM31

C	−1.90083	3.671212	0.158263
C	−3.22975	3.29643	0.230081
C	−3.58024	1.963667	0.113748
C	−2.62296	0.977208	−0.07302
C	−1.29097	1.378089	−0.1314
C	−0.92851	2.707676	−0.0262
C	−2.95206	−0.49981	−0.09688
C	−2.1958	−1.27838	−1.09704
C	−2.91459	−1.09881	1.24789
C	−1.70901	−2.52871	−0.83422
C	−2.42621	−2.34832	1.473787
C	−1.8278	−3.08611	0.436869
H	−1.43929	−4.07274	0.628124
H	−3.37651	−0.52396	2.03571
H	−1.61765	4.708602	0.242391
H	−2.48767	−2.77415	2.464127
H	0.117472	2.965904	−0.08943
H	−3.99933	4.038784	0.369818
H	−4.61786	1.678601	0.156329
H	−0.66755	−0.86893	2.139908
Si	3.097114	−0.03061	0.140352
O	4.515372	−0.52818	0.859985
O	3.983064	0.485071	−1.17478
Si	0.823215	−0.04305	0.717948
O	1.865651	−1.09531	−0.06878
O	2.106318	1.020396	0.91809
O	0.225671	−0.51506	2.128487
Si	5.382422	−0.00964	−0.44744
O	6.835939	0.005475	−0.8223
Cl	−4.76494	−0.70006	−0.6779
H	−1.20516	−3.07444	−1.6188
O	−0.30895	0.445653	−0.33096
O	−2.08434	−0.66143	−2.26973
H	−1.57858	−1.18817	−2.88743

IM32

H	1.111278	−0.89737	3.625396
Si	2.27328	−0.05927	0.211725
O	3.726748	−0.87469	0.139165
O	2.669381	0.709295	−1.21141
Si	0.443225	−0.01735	1.694091
O	0.871719	−0.90101	0.331631
O	1.904982	0.807925	1.55388

O	0.316231	−0.75837	3.11667
Si	4.10482	−0.10643	−1.27539
O	5.268507	−0.14287	−2.22339
C	−2.26304	−1.49682	0.234556
C	−1.82207	−2.0653	−0.95268
C	−1.03438	−3.20153	−0.92764
C	−0.69401	−3.78252	0.278356
C	−1.15008	−3.23157	1.462742
C	−1.93575	−2.09459	1.438311
Cl	−2.2651	−1.38373	−2.46927
H	−0.69839	−3.62315	−1.86127
H	−0.07637	−4.66655	0.288523
H	−0.89126	−3.67724	2.409964
H	−2.29553	−1.63816	2.347071
C	−2.61976	0.804621	−0.16248
C	−3.32262	1.474229	−1.14437
C	−2.93129	2.738361	−1.53786
C	−1.83779	3.34435	−0.94855
C	−1.1489	2.680375	0.047362
C	−1.53075	1.408619	0.456706
H	−4.16662	0.975689	−1.59171
H	−3.47749	3.25447	−2.31086
H	−1.51737	4.330109	−1.24217
Cl	0.170046	3.451157	0.837134
O	−0.9353	0.803466	1.512046
O	−3.08831	−0.40759	0.254975

IM33

C	2.991959	−1.97408	1.680289
C	2.460537	−1.1526	0.707864
C	1.591197	−1.67207	−0.24316
C	1.230282	−3.00385	−0.1976
C	1.753194	−3.82245	0.788687
C	2.639839	−3.31276	1.720849
C	0.889931	0.89861	1.837993
C	1.875875	1.088446	0.884337
C	−0.03322	1.898014	2.089807
C	1.927399	2.283862	0.179745
C	0.0235	3.090581	1.391186
C	1.005094	3.280223	0.434944
H	1.062948	4.199302	−0.12566
H	0.85766	−0.03319	2.381539
H	1.472024	−4.86313	0.818258

H	−0.79623	1.735834	2.834687
H	3.678536	−1.54663	2.393717
Cl	3.144515	2.51738	−1.01291
H	−0.69181	3.874002	1.585084
H	3.056349	−3.95295	2.482026
H	−0.76327	0.085836	−3.93257
Si	−2.22673	−0.23625	−0.52371
O	−3.84298	0.040983	−0.83272
O	−2.57606	−0.25896	1.105404
Si	−0.28401	−0.50303	−1.82299
O	−1.4752	−1.52391	−1.21013
O	−1.10764	0.791573	−1.13901
O	−0.12365	−0.39709	−3.41607
Si	−4.17313	0.022396	0.786264
O	−5.40541	0.197973	1.625386
H	5.751979	0.234678	−0.31858
H	0.549861	−3.39088	−0.94002
O	2.833701	0.15613	0.635459
O	1.17298	−0.84587	−1.23865

IM34

C	−1.18101	3.879075	−0.64626
C	−2.43324	4.040942	−0.08442
C	−3.14697	2.929447	0.324197
C	−2.63513	1.645369	0.195175
C	−1.37167	1.493712	−0.37894
C	−0.65996	2.608149	−0.80227
C	−3.46024	0.487749	0.736498
C	−3.68962	−0.62474	−0.25136
C	−2.92566	0.016509	2.048682
C	−3.50797	−1.92823	0.119659
C	−2.74801	−1.29396	2.334128
C	−3.03648	−2.29301	1.384597
H	−2.89424	−3.33476	1.614847
H	−2.75187	0.779267	2.794861
H	−0.61078	4.734751	−0.97203
H	−2.39331	−1.59195	3.310031
H	0.311186	2.46987	−1.25175
H	−2.85764	5.025871	0.032283
H	−4.44985	0.923808	0.932593
Cl	−3.81479	−3.21801	−0.99365
S	−4.21587	−0.05415	−1.80374
H	−4.42607	−1.25321	−2.34926

H	−4.12908	3.055461	0.75695
S	−0.65571	−0.12082	−0.66524
H	−0.57333	0.147223	2.371216
Si	3.175575	−0.1698	0.17565
O	4.591767	−0.19509	1.023669
O	4.005902	−0.3878	−1.23461
Si	0.894133	0.068825	0.72952
O	1.960906	−1.21514	0.575645
O	2.146419	1.112469	0.330701
O	0.345824	0.374853	2.217583
Si	5.458076	−0.4215	−0.39456
O	6.28202	−1.79778	−0.54291
H	5.809105	−2.62486	−0.56092
O	6.522926	0.742993	−0.7197
H	6.303133	1.646736	−0.51273

IM35

C	1.252753	3.532249	0.011699
C	2.544351	3.51112	−0.47295
C	3.235444	2.315446	−0.55519
C	2.660404	1.114053	−0.16567
C	1.345279	1.145488	0.318802
C	0.662521	2.35012	0.414826
C	3.373694	−0.22031	−0.32686
C	3.417302	−1.05896	0.895015
C	2.900518	−0.9425	−1.52295
C	3.274017	−2.42526	0.818712
C	2.734034	−2.28643	−1.53781
C	2.938801	−3.04967	−0.37107
H	2.81585	−4.12007	−0.39786
H	2.792869	−0.34653	−2.41763
H	0.704057	4.45809	0.083271
H	2.450372	−2.78143	−2.45472
H	−0.34513	2.348139	0.799593
H	3.026464	4.423477	−0.78726
S	3.739854	−0.20257	2.371936
H	3.736918	−1.28234	3.155853
H	4.244793	2.314004	−0.92728
S	0.493433	−0.31902	0.899746
H	0.57791	−0.88175	−2.10425
Si	−3.27278	−0.20555	−0.18331
O	−4.64404	−0.31115	−1.09483
O	−4.18043	0.060978	1.169315

Si	−0.95761	−0.35882	−0.61146
O	−2.17357	−1.43853	−0.20282
O	−2.09281	0.874773	−0.59556
O	−0.31317	−0.52931	−2.08213
Si	−5.58992	−0.03433	0.262958
O	−6.59526	−1.20873	0.714693
H	−6.24002	−2.04886	0.990184
O	−6.50099	1.291328	0.17905
H	−6.15714	2.067816	−0.25314
Cl	5.219356	0.103978	−0.70846
H	3.383058	−3.01802	1.714343

IM36

H	3.211366	0.006727	−3.63519
Si	1.56762	1.384852	−0.51802
O	1.699755	3.023254	−0.66979
O	0.736099	1.571327	0.896012
Si	2.128976	−0.56586	−1.7409
O	0.867922	0.545751	−1.7571
O	2.88054	0.391853	−0.5749
O	2.919094	−0.75155	−3.1356
Si	0.801099	3.236558	0.734964
C	−2.65575	−0.42063	−0.73129
C	−3.88376	−0.60447	−0.102
C	−4.80744	0.426944	−0.03522
C	−4.51738	1.649201	−0.60964
C	−3.30778	1.844154	−1.25696
C	−2.38807	0.813393	−1.31641
Cl	−4.29151	−2.11099	0.626575
H	−5.74861	0.259081	0.462708
H	−5.2449	2.444395	−0.56073
H	−3.09292	2.787282	−1.73694
H	−1.44722	0.932683	−1.83122
C	−0.45633	−1.50461	0.499858
C	−0.98106	−1.04595	1.699217
C	−0.18354	−0.95177	2.819579
C	1.142297	−1.33257	2.775613
C	1.671933	−1.79268	1.586446
C	0.893329	−1.87439	0.430368
H	−2.01829	−0.76321	1.757041
H	−0.60355	−0.5867	3.743681
H	1.771107	−1.27472	3.648324
Cl	3.328713	−2.25808	1.574907

S	−1.46516	−1.71349	−0.93616
S	1.599486	−2.4771	−1.09075
O	−0.66398	3.895059	0.595614
H	−1.38202	3.328024	0.320821
O	1.575661	4.091182	1.859305
H	2.526236	4.037536	1.906599

IM37

C	−1.95581	3.582566	0.260631
C	−1.4554	2.383883	−0.2318
C	−0.19543	2.367	−0.82825
C	0.538218	3.540131	−0.92952
C	0.034608	4.728137	−0.43685
C	−1.21475	4.744111	0.158773
C	−4.61039	−2.1831	−1.41382
C	−4.79807	−1.2873	−0.40093
C	−5.54636	−3.20699	−1.52104
C	−5.82134	−1.32076	0.500243
C	−6.60178	−3.28104	−0.62567
C	−6.74947	−2.34626	0.385905
H	−7.57089	−2.40336	1.083259
H	−3.77941	−2.10256	−2.09722
H	0.614505	5.63359	−0.5175
H	−5.446	−3.94336	−2.30352
H	−2.93073	3.608248	0.722848
Cl	−5.96019	−0.12907	1.743974
H	−7.3237	−4.07763	−0.71292
H	−1.62019	5.665617	0.546624
S	0.460002	0.867148	−1.53452
S	−2.35757	0.87241	−0.12584
H	−3.34596	1.348336	0.635678
H	1.509782	3.506906	−1.39672
Si	3.616507	−0.65882	0.203801
O	2.891843	0.775155	0.582099
O	2.149921	−1.31835	−0.17586
Si	5.846716	−1.44869	0.193124
O	4.83458	−0.69174	−0.91048
O	4.592709	−1.40387	1.30624
Si	1.393096	0.129962	0.20032
O	7.171778	−0.64643	0.635025
H	7.08422	0.193825	1.075691
O	6.370871	−2.90381	−0.25722
H	5.767027	−3.50888	−0.67812

O	0.427741	0.145692	1.488717
H	−0.52259	0.150615	1.344439
